# CLICK-17, a DNA enzyme that harnesses ultra-low concentrations of either Cu^+^ or Cu^2+^ to catalyze the azide-alkyne ‘click’ reaction in water

**DOI:** 10.1093/nar/gkaa502

**Published:** 2020-06-10

**Authors:** Kun Liu, Prince Kumar Lat, Hua-Zhong Yu, Dipankar Sen

**Affiliations:** Dept. of Chemistry, Simon Fraser University, Burnaby BC V5A 1S6, Canada; Dept. of Molecular Biology & Biochemistry, Simon Fraser University, Burnaby BC V5A 1S6, Canada; Dept. of Chemistry, Simon Fraser University, Burnaby BC V5A 1S6, Canada; Dept. of Molecular Biology & Biochemistry, Simon Fraser University, Burnaby BC V5A 1S6, Canada; Dept. of Chemistry, Simon Fraser University, Burnaby BC V5A 1S6, Canada; Dept. of Molecular Biology & Biochemistry, Simon Fraser University, Burnaby BC V5A 1S6, Canada

## Abstract

To enable the optimal, biocompatible and non-destructive application of the highly useful copper (Cu^+^)-mediated alkyne-azide ‘click’ cycloaddition in water, we have isolated and characterized a 79-nucleotide DNA enzyme or DNAzyme, ‘CLICK-17’, that harnesses as low as sub-micromolar Cu^+^; or, surprisingly, Cu^2+^ (without added reductants such as ascorbate) to catalyze conjugation between a variety of alkyne and azide substrates, including small molecules, proteins and nucleic acids. CLICK-17’s Cu^+^ catalysis is orders of magnitude faster than that of either Cu^+^ alone or of Cu^+^ complexed to PERMUT-17, a sequence-permuted DNA isomer of CLICK-17. With the less toxic Cu^2+^, CLICK-17 attains rates comparable to Cu^+^, under conditions where both Cu^2+^ alone and Cu^2+^ complexed with a classic accelerating ligand, THPTA, are wholly inactive. Cyclic voltammetry shows that CLICK-17, unlike PERMUT-17, powerfully perturbs the Cu(II)/Cu(I) redox potential. CLICK-17 thus provides a unique, DNA-derived ligand environment for catalytic copper within its active site. As a *bona fide* Cu^2+^-driven enzyme, with potential for being evolved to accept only designated substrates, CLICK-17 and future variants promise the fast, safe, and substrate-specific catalysis of ‘click’ bioconjugations, potentially on the surfaces of living cells.

## INTRODUCTION

The copper (Cu^+^)-dependent azide-alkyne cycloaddition (‘CuAAC’) reaction is perhaps the most versatile and widely used of the ‘click’ chemistries used for bioconjugations, and more generally for coupling together two molecules of interest, in this case, a terminal alkyne and an organic azide (1–3). The CuAAC reaction has found many applications, including bioconjugations involving living cells. Powerful and versatile as it is, the requirement for Cu(I) (Cu^+^) ions in CuAAC can be problematic, owing to the concomitant generation of destructive reactive oxygen species (ROS) by Cu(I) (4–6). This, and the desirability for ∼100 μM–1 mM concentrations of Cu(I) (toxic to most living cells), have inhibited the full-blown use of CuAAC in biological applications. A number of innovative approaches to minimize or abrogate the toxic effect of Cu(I), and of copper in general, have been developed in recent years. These include (i) utilization of specific ligands to bind and stabilize Cu(I); such ligands, including the water-soluble tris(3-hydroxypropyltriazolylmethyl)amine (THPTA), accelerate the reaction by perturbing the Cu(II)/Cu(I) redox potential toward Cu(I) as well as serving as sacrificial oxidation substrates for the generated ROS (7–9); (ii) developing Cu(I)-chelating azides as participating reagents (10) and (iii) the use of enforcedly proximal azide and alkyne (11) or strained alkynes (12,13), both under copper-free conditions. These novel approaches afford substantial benefits; nevertheless, they continue to show collateral disadvantages—for instance, the relatively promiscuous reactivity (such as with thiols) of the strained alkynes used for copper-free AAC (14).

Biological transformations of any kind are most optimally facilitated by enzymes. If there were to exist an enzyme (protein or nucleic acid) capable of catalyzing the azide-alkyne cycloaddition (AAC) reaction with high efficiency under low-to-zero copper concentrations, it should prove to be a highly useful reagent for catalyzing this powerful coupling reaction, especially in biological contexts. However, AAC or CuAAC are not metabolic reactions *per se*; and out of the large number of naturally occurring copper-utilizing proteins, it is difficult to identify an obvious candidate that could be ‘evolved’ towards catalyzing CuAAC. Nevertheless, powerful methodologies exist, such as *in vitro* selection from vast, random sequence, single-stranded RNA or DNA libraries (‘SELEX’) (15–17), for *de novo* identification of RNA or DNA biocatalysts (ribozymes and DNAzymes) suitable for catalyzing even non-metabolic reactions (18–22).

**Figure 1.**
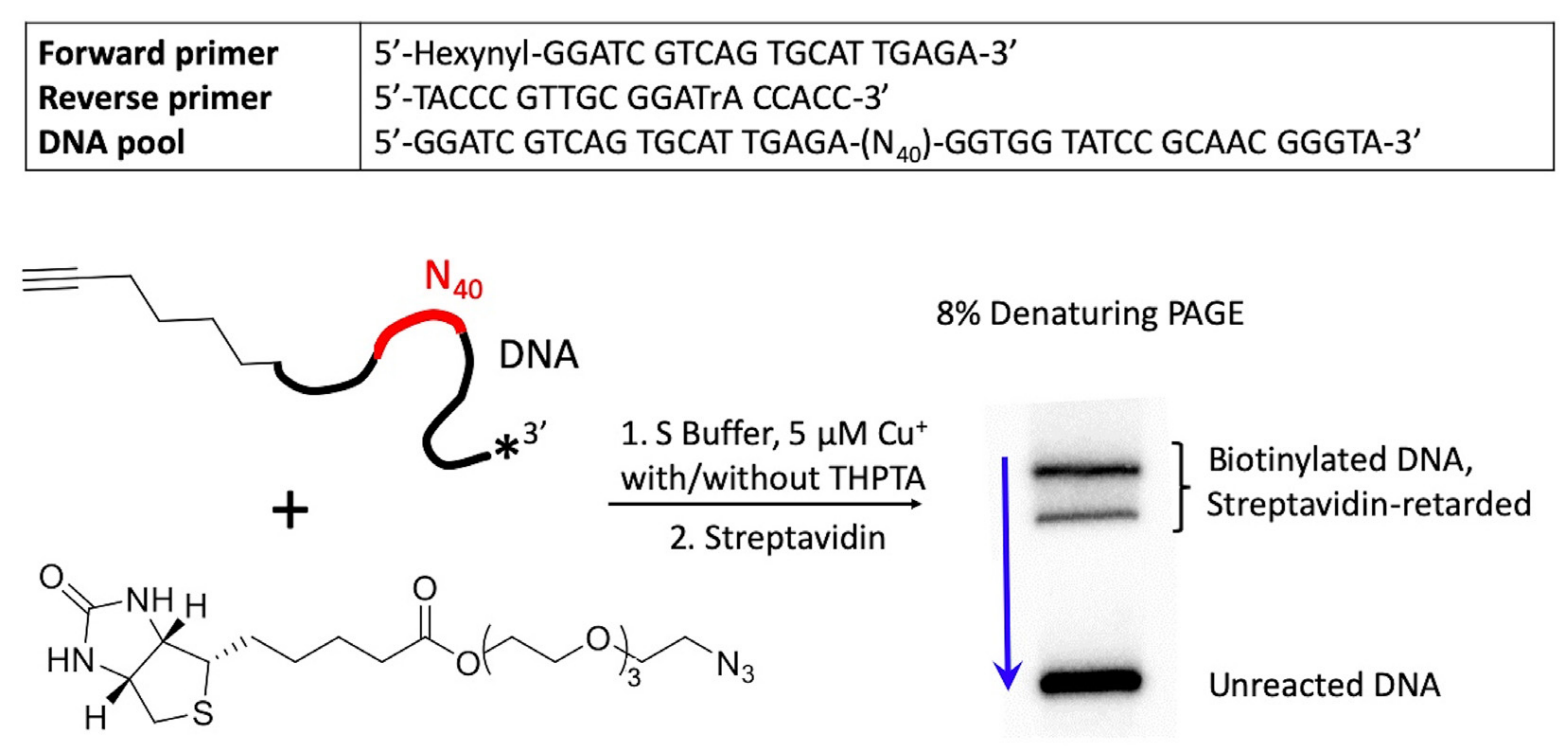
Design of an *in vitro* selection protocol for a CuAAC-catalyzing DNAzyme. The ‘N_40_’ region shown in red indicates the location of the randomized stretch of DNA sequence present within each individual DNA molecule (∼10^14^ total such molecules within the starting selection ‘library’). The blue arrow (right) shows the direction of electrophoretic migration of DNA in a denaturing polyacrylamide gel (PAGE).

Currently, standard conditions for CuAAC in water involve generation of Cu(I) *in situ* via reduction of 100 μM–1 mM of an added Cu(II) salt by a reducing agent (commonly, ascorbate), generally in the presence of a 5-fold excess (over copper) of a ligand such as THPTA (23–27). We hypothesized that efficient catalytic DNAs capable of harnessing very low Cu(I) concentrations could, in principle, be identified despite the propensity of Cu^2+^ and Cu^+^ for non-specific binding to DNA (to the backbone phosphodiesters as well as to sites on the heterocyclic nucleobases) (28–30). Figure 1 shows the design of a randomized DNA library for our selection, consisting of ∼10^14^ distinct 80-nt sequences, each incorporating 40 random deoxynucleotides (‘N_40_’) flanked on either side by 20-nt fixed sequences suitable for primer-binding for PCR-amplification (Figure 1; Materials and Methods).

Herein, we report the selection of a highly copper-efficient catalytic DNA (DNA enzyme, deoxyribozyme, or DNAzyme), ‘CLICK-17’, which catalyzes CuAAC *in cis* with as little as 50–200 nM Cu^+^. Such an initial, single-turnover version of CLICK-17 (operating ‘*in cis*’, as 5′-hexynyl-CLICK-17 or ≡-CLICK-17) was then converted to a true enzyme, catalyzing multiple turnovers of a variety of small molecule as well as macromolecule substrates *‘in trans’* (22). The most intriguing result we report is that CLICK-17 is catalytic with *either* Cu^+^ or Cu^2+^ as added cofactors (the latter in the absence of added ascorbate). The Cu^2+^ reaction is optimal in the 5–20 μM copper ion concentration range; under which conditions, *neither* a sequence-permuted isomer of CLICK-17 (‘PERMUT-17’) DNA *nor* a 5-fold excess THPTA are found to support the CuAAC reaction at any level.

## MATERIALS AND METHODS

### Chemicals

HEPES (Sigma-Aldrich, 99.5% purity, copper ≤ 5 ppm); MgCl_2_.H_2_O (Sigma-Aldrich, 99% purity, copper ≤ 5 ppm); LiOH·H_2_O (Sigma-Aldrich, ≥ 99% purity, copper ≤ 5 ppm); sodium ascorbate (Sigma-Aldrich, 98% purity); NaCl (Sigma-Aldrich, 99% purity); KCl (ACP Chemicals, 99%); sodium acetate (Bio Basic, 99% purity); 5-hexyn-1-ol (Sigma-Aldrich, 96% purity); propargyl alcohol (Sigma-Aldrich, 99% purity); 3-azido-7-hydroxycoumarin (AK Scientific 98% purity); azide-PEG3-biotin conjugate (Sigma-Aldrich, purity not specified by the manufacturer); NaOH (Sigma-Aldrich, 99% purity); CuSO_4_ (Fisher Scientific, 99% purity); Azido-PEG2-NHS ester (BroadPharm, 98% purity); Fluorescein-NHS ester (BroadPharm, 95% purity); AFDye 546 (Click Chemistry Tools, ≥95%); Lysozyme, from chicken egg white (Sigma-Aldrich, ≥ 98% purity); Nuclease P1 (NEB, 100,000 Units/mL).

### DNA and 3′ end-labeling

An 80-deoxynucleotide (80-nt) single-stranded DNA library (5′-GGATC GTCAG TGCAT TGAGA *N_40_*GGTGG TATCC GCAAC GGGTA) was designed to contain a central stretch of 40 randomized deoxynucleotides (‘N_40_’) flanked by two fixed, 20-nt primer binding sequences (Core DNA Services Inc., Calgary, Canada). All other DNAs, modified or unmodified, were from Integrated DNA Technologies (Illinois, USA). Oligonucleotides were size-purified using denaturing gel-electrophoresis, followed by ethanol precipitation(s) and scrupulous 70% aqueous ethanol washes to remove residual urea.

The 5′-hexynyl moiety was linked to the ‘forward’ PCR primer sequence (5′-hexynyl-GGATC GTCAG TGCAT TGAGA-3′) during automated DNA synthesis. The reverse primer had the sequence: 5′-TACCC GTTGC GGrATA CCACC-3′ (where ‘rA’ shows the location of a single ribonucleotide placed within the DNA oligonucleotide). Azide-PEG_3_-biotin (Sigma) was chosen as a substrate based on its solubility in water and because, following CuAAC, azide-clicked 5′-hexynyl-DNA molecules would be tagged with biotin, enabling their separation from unreacted DNA *via* binding to streptavidin (Figure 1).

For 3′-end radioactive labelling, 50 pmol of a given oligonucleotide was dissolved in 10 mM Li-HEPES·(pH 7.4) buffer and denatured by boiling for 5 min. The cooled DNA was then 3′-^32^P labeled using a standard protocol for use of Terminal Deoxynucleotidyl Transferase (Thermo Fisher Scientific, Vilnius, Lithuania). 1 μl of [α-^32^P]-dCTP (Perkin-Elmer) was added, and the reaction mixture was incubated for 1 h at 37 °C. The 3′-^32^P labeled oligonucleotide was then size-purified in 8% denaturing gels containing 7 M urea.

### *In vitro* selection

5 μM Cu^+^ was the concentration chosen for the first round of *in vitro* selection, to encourage selection of DNAzymes that worked with ultra-low Cu^+^ concentrations or required it not at all. 500 pmol of the DNA library was amplified by a large-scale (20 ml) PCR in order to incorporate the ‘forward’ 5′-hexynyl primer in one of the two strands. The ‘reverse’ primer contained an internal riboside nucleotide. 2.5 ml PCR reactions were used for round 2 and for all subsequent rounds of selection.

5′-Hexynyl-labeled single stranded DNA was recovered following NaOH-catalyzed ribonucleotide cleavage of one of the two component strands of the DNA duplexes, and then size-purification of the 5′-hexynyl-modified strand away from the cleaved complementary strand by preparative denaturing polyacrylamide gel electrophoresis.

The entirety of the recovered 5′-hexynyl labeled ssDNA pool, doped with a portion of the same strand pool 3′-labeled with ^32^P using terminal transferase (ThermoFisher Scientific, Vilnius, Lithuania) was used for the first round of *in* vitro selection, with parallel selections being performed with and without added THPTA (25 μM). The 5′-hexynyl labeled ssDNA was first heated to 100°C in S buffer (50 mM HEPES, pH 7.4, 300 mM NaCl, 50 mM KCl, 20 mM MgCl_2_), then cooled down to 22°C over a period of 10 min in a thermal cycler. Azide-PEG3-biotin (‘azide-biotin’) to a final concentration of 2.5 mM was then added, followed by CuSO_4_ to 5 μM. The CuAAC reaction was initiated at 22°C by addition of sodium ascorbate to 2.5 mM. The DNA solution was incubated at 22°C in a thermal cycler for 2 h, following which the DNA was ethanol precipitated, washed with 70% ethanol, air-dried, and dissolved in 5 μl of 10 mM HEPES buffer, pH 7.4 containing ∼1.9 nmol dissolved streptavidin. The solution was loaded directly onto a denaturing gel (8% acrylamide, 7 M urea), and the streptavidin-retarded DNA bands, visualized by phosphorimagery (Typhoon 9410 Phosphorimager, Amersham Biosciences), were excised from the gel, eluted into TE buffer (10 mM Tris, pH 7.4, 0.1 mM EDTA) and collected by ethanol precipitation. The recovered DNA was PCR amplified, again using one 5′-hexynyl labeled primer and one internal ribonucleotide containing primer, followed by base treatment and gel purification (as above), to generate an enriched pool of 5′-hexynyl labelled ssDNA for next round of *in vitro* selection. Following 25 rounds of selection, the enriched DNA sequences were cloned into *Escherichia coli* and 40 clones picked for sequencing (Genewiz, Canada). Preliminary CuAAC experiments were done with individual DNA clones (all 5′-hexynyl labeled), and clone CLICK-17, 79-nt long, was picked as a promising catalytic candidate. CLICK-17 had the nucleotide sequence: 5′-GGATC GTCAG TGCAT TGAGA *TTATTATGCAACTCTATGGGTCCACTCTGTGAATGTGAC* G GTGGT ATCCG CAACG GGTA-3′. A sequence-permuted variant of CLICK-17 DNA, named ‘PERMUT-17’, was used as a control DNA throughout; PERMUT-17 had the nucleotide sequence: 5′-GGATC GTCAG TGCAT TGAGA GACAT ACATG TTATC GGTAT GTTCG AGCTC TATAT CTCGT ACCCG TTGCG GATAC CACC.

### *In cis* reactions

5′-Hexynyl-CLICK-17 DNA (‘≡-CLICK-17’) or 5′-hexynyl-PERMUT-17 DNA (‘≡-PERMUT-17’), either 3′-^32^P-labeled or not, were denatured and refolded in R buffer (50 mM Li-HEPES, pH 7.4, 20 mM MgCl_2_) by boiling the solution and cooling down to 22°C, over 10 min, in a thermal cycler. 2.5 mM azide-PEG3-biotin (‘azide-biotin’) was then added. To start the reaction, a mixture of CuSO_4_ and sodium ascorbate in 10 mM Li-HEPES, pH 7.4 was added. For Cu(II)-catalyzed reactions, only CuSO_4_ in 10 mM Li-HEPES, pH 7.4 was added. Following incubation at 22°C in a thermal cycler for the indicated reaction time, the DNA was ethanol precipitated, washed thoroughly with cold 70% ethanol, air-dried, redissolved and treated with streptavidin (2 μl from a 5 mg/ml stock). The resulting solutions were loaded directly onto a denaturing 10% polyacrylamide gel. DNA bands in the gel following electrophoresis were visualized either by phosphorimagery or by ethidium bromide staining followed by fluorescence imagery (fluorescent gel images were recorded with a GENi gel imaging system, Syngene, Frederick, Maryland).

For *in cis* reactions of lysozyme-(N_3_)_n_ (see below) with ≡-CLICK-17, 1 μM lysozyme-(N_3_)_n_ was mixed with 2 μM ≡-CLICK-17 DNA, along with copper salts (or not) with or without ascorbate, as indicated in the text. Reactions were carried out in R buffer, at 22°C, for 2.5 h.

### *In trans* reactions

2 μM DNA oligonucleotides were refolded in R buffer. 50 μM 3-azido-7-hydroxycoumarin (‘azide-coumarin’, Jena Bioscience, Germany), from a fresh stock made in dimethylsulfoxide, was then added followed by a mixture of appropriate concentrations of CuSO_4_ and sodium ascorbate in 10 mM Li-HEPES, pH 7.4 (for the Cu(I) reactions). For Cu(II) reactions, CuSO_4_ alone (and, no ascorbate) in 10 mM Li-HEPES, pH 7.4, was added. The solutions were transferred to the wells of a 384-well Falcon plate (black, flat bottomed, from Corning) and incubated at 22°C for 5 min. 5-Hexyn-1-ol (‘hexynol’) or propargyl alcohol was finally added to initiate the reaction. End-point fluorescence readings were carried out in a fluorescence plate reader (Infinite M200 Pro, Tecan). Excitation was at 403 nm, and emission was monitored at 480 nm.

For *in trans* reaction of lysozyme-(N_3_)_*n*_ (see below) with alkynated Alexa Fluor Dye 546 (≡-AFDye 54) catalyzed by CLICK-17, 20 μM lysozyme-(N_3_)_n_ was mixed with 200 μM ≡-AFDye 546, 4 μM folded CLICK-17 DNA, along with copper salts (or not) with or without ascorbate, as indicated in the text. Reactions were carried out in R buffer, at 22°C, for 2.5 h.

### Lysozyme labeling with Azido-PEG2-NHS Ester

5 mg chicken egg lysozyme (Sigma-Aldrich) was dissolved in 950 μl of 0.1 mM NaHCO_3_ buffer, pH 8.3. 1 mg azide-PEG2-NHS ester (BroadPharm, San Diego) was dissolved separately in 50 μl DMSO. The two solutions were mixed together and incubated for 2 h at 22°C with gentle rotation of the tube. 50 mM ethanolamine was then added and the reaction solution incubated for a further 0.5 h at 22°C to quench unreacted NHS ester. The reaction mixture was then passed through Mirocon-10k centrifugal filters (10k Da size cut-off, Cedarlane, Burlington). Two further washes, each with 400 μl ddH_2_O, were carried out. The concentrated protein was dissolved in about 100 μl 10 mM HEPES buffer (pH 7.4). The concentration of protein was determined by the BCA assay (BCA protein assay kit, Thermo Fisher Scientific, Toronto), and mass spectrometry was carried out to verify the azide-labeling.

### Mass spectrometry

An Agilent 1200 HPLC couple to a Bruker maXis Impact Ultra-High Resolution tandem TOF (UHR-Qq-TOF) mass spectrometer was used. The software used was: Compass 1.5. The ionization mode used was positive electrospray ionization (+ESI), with gas temperature (180°C); Gas Flow (8 l/min); nebulizer (2 bar); capillary voltage (4200 V); mass range (300–2500 Da). The calibrant used was: Agilent Tune Mix L. For HPLC, the column used was: Zorbax 300SB-C8 particle size 3.5 micron, 50 mm length × 2.1 mm diameter (Agilent Technologies). The column temperature was maintained at 30°C. The HPLC Gradient Table is given below:

Solvent A: Water with 0.1% formic acid

Solvent B: Acetonitrile with 0.1% formic acid

**Table:** 

Time (min)	% Solvent B	Flow rate (ml/min)
0	2	0.3
1	2	0.3
3	30	0.3
13	60	0.3
13.1	60	0.5
15	60	0.5
15.1	2	0.5
20 (stop)	2	0.5

### Mass spectrometry of *in cis* ≡-CLICK-17 DNA-labeled lysozyme

Mass spectrometry was carried out in a Bruker maXis Impact Quadrupole Time-of-Flight LC/MS System. For unconjugated lysozyme-(N_3_)_*n*_, 1 mg/ml solution was prepared in ddH2O, and loaded directly. For ≡-CLICK-17/lysozyme-(N_3_)_*n*_ conjugates, the DNA was first cleaved by Nuclease P1 (New England Biolabs, MA, USA) to leave one guanosine (the 5′-most deoxyguanosine of the CLICK-17 sequence) per conjugated DNA still attached to lysozyme.

For mass-spectrometry, *in cis* conjugation of ≡-CLICK-17 and lysozyme-(N_3_)_*n*_, 2 μM ≡-CLICK-17 DNA, 2 μM lysozyme-(N_3_)_*n*_, were reacted together at 22°C for 1 h in R Buffer supplemented with 4 μM CuSO_4_ and 100 μM sodium ascorbate. The reaction mixture was passed through Microcon-10K centrifugal filter to desalt. The concentrated solution was diluted with ddH_2_O, supplemented with 1/10 volume of 10× nuclease P1 reaction buffer, and 0.1 μl nuclease P1 per 50 μl reaction. Digestion was carried out at 37°C for 15 min, and the reaction quenched by heating at 75°C for 10 min. The resulting solution was cleaned up by passing through a Microcon-10K centrifugal filter. After adjusting the sample volume to make the concentration of protein ∼1 mg/ml, samples were analyzed by mass spectrometry.

### Cyclic voltammetry

The buffer solution for all CV measurements was: 20 mM HEPES, pH 7.4, and 20 mM MgCl_2_ (R Buffer). The dissolved DNA was folded as described above. Prior to making CV measurements, CuSO_4_ was added and rested for 5 min. All samples was degassed with argon. Cyclic voltammetry measurements were carried out in a three-electrode, single chamber glass cell with a CHI 1040A Electrochemical Analyzer (Austin, TX). A platinum wire and an Ag|AgCl|3 M NaCl electrode were used as counter electrode and reference electrode, respectively. A glassy carbon electrode was used as the working electrode. All CV measurements were performed in a Faraday cage at 22°C, in R Buffer, which had been subjected to deoxygenation for at least 15 min. The scan rate for all CV measurements was maintained at 50 mV/s.

## RESULTS

### DNAzyme design and selection

The key design strategy for identifying catalytic DNA sequences out of a large random-sequence library is to select first for quasi-catalytic DNAs, that carry out a single catalytic turnover (i.e. work ‘*in cis*’); and are in the process chemically modified (tagged) such that they can be sequestered away from non-active DNAs within the library. Our single-stranded DNA (ssDNA) pool was synthesized with a 5′-hexynyl attachment on each DNA (Figure 1). These sequences were allowed to fold to form their distinctive secondary/tertiary structures in an aqueous buffer solution (‘S Buffer’: 50 mM HEPES, pH 7.4, 300 mM NaCl, 50 mM KCl, 20 mM MgCl_2_), then supplemented with 5 μM Cu^+^ and freely diffusible azide-biotin for 2 h at 22°C (Figure 1). DNA sequences capable of catalyzing CuAAC in this very low-copper regime self-tagged with biotin, and were separated on that basis from the unreacted DNAs (the biotin ‘tag’ binds tightly to the protein streptavidin, which in turn retards the electrophoretic mobility of biotinylated DNAs in a gel). Supporting Figure S1 provides a schematic diagram for the entire SELEX procedure.

A total of 25 rounds *in vitro* selection were carried out. Two parallel selection experiments were conducted, one with Cu^+^ as the catalytic cofactor, and another with THPTA/Cu^+^ as the cofactor. The enrichment of CuAAC-active DNAs through the selection rounds could be tracked by monitoring the overall conversion percentage of pool DNA in rounds 1–7 (Supporting Figure S2). The enrichment trends observed for both selections were roughly comparable; by round 7, > 50% of the input 5′-hexynyl-DNA had reacted with the input azide-biotin within 2 h. By Round 25, pool enrichment had stabilized. Forty clones of the Round 25 pool were picked and sequenced, and the results are compiled in Supporting Figure S3. A number of the sequenced clones showed identical sequences, and five families of sequence were identified. Clones were picked from each family and prepared as 5′hexynyl-labeled-ssDNA (‘≡-DNA’), then tested for *in cis* CuAAC-promoting activities. Testing was carried out in S buffer, used for the *in vitro* selection, and clone CLICK-17 was picked as a promising catalytic candidate. 5′-hexynyl-CLICK-17 (‘≡-CLICK-17’) was then studied intensively. It was found that ≡-CLICK-17’s reaction kinetics in a relatively simple reaction buffer (‘R buffer’: 50 mM Li-HEPES, pH 7.4, 20 mM MgCl_2_) were comparable to those in the higher ionic-strength S buffer. Consequently, all detailed characterization studies on ≡-CLICK-17 were carried out, except where explicitly stated to be otherwise, in R buffer.

### The *in cis* reaction of 5′-hexynyl-CLICK-17 DNA (≡-CLICK-17)

**Figure 2.**
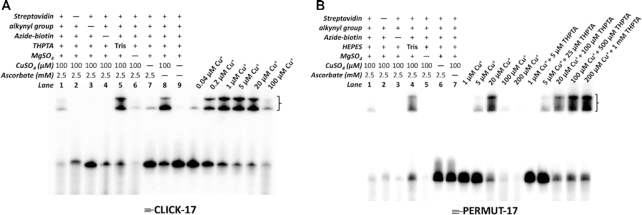
*In cis* catalysis by 5′-hexynyl-CLICK-17 DNA (≡-CLICK-17). Panels A and B: 5′-hexynyl-CLICK-17 DNA (≡-CLICK-17) catalyzes CuAAC *in cis* in R buffer (**A**); by contrast, 5′-hexynyl-PERMUT-17 DNA (≡-PERMUT-17) does not catalyze the reaction (**B**). Reaction conditions were: 2 μM ≡-CLICK-17 (or ≡-PERMUT-17) and 2.5 mM azide-biotin in R buffer, to which 100 μM CuSO_4_ (the default concentration, unless indicated), and 2.5 mM sodium ascorbate were added. Reactions proceeded at 22°C for 1 h except in the copper titration lanes (where they proceeded for 30 min).

Figure 2a shows the CuAAC capabilities of 3′-^32^P-labeled ≡-CLICK-17 DNA under different reaction conditions. ≡-CLICK-17 was heat-denatured and refolded in R buffer. The generic *in cis* reaction with azide-biotin consisted of 2 μM folded ≡-CLICK-17, to which was added 100 μM CuSO_4_, 5 mM azide-biotin and 2.5 mM of sodium ascorbate. These solutions were incubated at 22°C for 1 h for the various control experiments (first nine lanes from the left in Figure 2A), and for 0.5 h for the copper titration (0.04–100 μM Cu^+^) experiments (Figure 2A). In all lanes (excepting lane 2), the reaction was terminated by ethanol precipitation, and the recovered and redissolved DNA was treated with streptavidin prior to loading into a non-denaturing PAGE gel. Identical values of ^32^P counts were loaded into each lane. Biotinylation of DNA gave rise to two or more DNA bands of retarded electrophoretic mobility by virtue of streptavidin binding (indicated by brackets in Figure 2A; the different retarded bands represent different stoichiometries of these robust complexes—one streptavidin protein is capable of binding up to four biotinylated DNAs).

Although precisely equal amounts of DNA (corresponding to ^32^P counts) were loaded in each lane, the different incubations show different levels of DNA in the different lanes of the gel. The first six lanes of the experiment show that in all incubations carried out with 100 μM Cu^+^, significant degradation of the ≡-CLICK-17 DNA occurs, particularly in the absence of THPTA, presumably via ROS generation in the solution (4–6). Substitution of Li-HEPES by Tris as the buffering reagent lowers this degradation (‘Tris buffer’ lane in Figure 2A). The presence of magnesium is required in the reaction buffer, presumably for the catalytically relevant folding of ≡-CLICK-17. The absence of magnesium ions also leads to a high level of ≡-CLICK-17 degradation; presumably, Mg^2+^ modulates the binding (and degradative effect) of Cu^+^ to the DNA. Curiously, the complete absence of copper (‘-CuSO_4_’) *does* generate a small amount of streptavidin-retarded product, presumably from very low levels of copper-free AAC catalyzed by the folded ≡-CLICK-17.

The most curious, yet reproducible, result is the high level of CuAAC product seen in the presence of 100 μM CuSO_4_ in the absence of any added ascorbate (‘- sodium ascorbate’, Figure 2A). This phenomenon was investigated in more depth, below.

The six lanes on the extreme right side of Figure 2A show the yield of the CuAAC product formed from ≡-CLICK-17 as a function of Cu^+^ concentration. With as little as 200 nM Cu^+^, ∼50% biotinylation of ≡-CLICK-17 is reached within 0.5 h; with 1 μM Cu^+^, conversion is ∼80% complete in this time frame. While DNA degradation is modest at ≤20 μM Cu^+^, at 100 μM Cu^+^, such degradation is heavy (as shown by the smears at the bottom of the gel).

To determine if the above results are specific to ≡-CLICK-17, as opposed to 5′-hexynylated ssDNAs in general, we carried out analogous experiments with a nucleotide-sequence permuted DNA isomer of ≡-CLICK-17 (‘≡-PERMUT-17’; nucleotide sequence given in Methods). Figure 2B shows these results. Here, again, Tris buffer generates less DNA degradation than does HEPES buffer. With ≡-PERMUT-17, unlike ≡-CLICK-17, however, (a) no biotinylation is evident with 100 μM CuSO_4_ in the absence of ascorbate and (b) quantitative biotinylation of ≡-PERMUT-17 requires ∼20 μM Cu^+^, between 20- and 100-fold higher than required for ≡-CLICK-17. The impact of THPTA (present at 5-fold > [Cu^+^]) on ≡-PERMUT-17 biotinylation is not majorly different from that in the absence of THPTA; only, at high copper concentrations (100 and 200 μM Cu^+^), THPTA reduces ≡-PERMUT-17 degradation.

**Figure 3.**
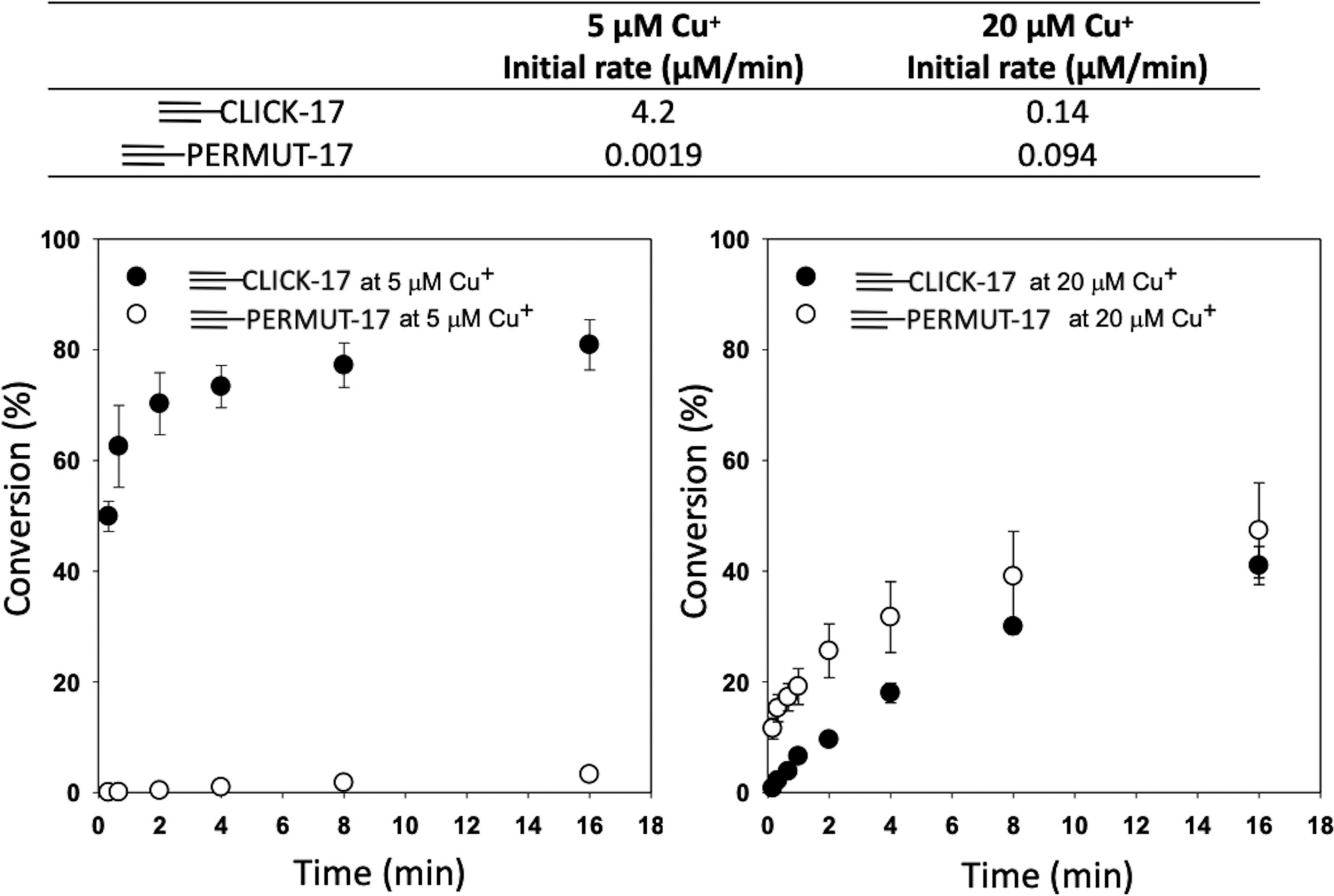
Plots of the reaction time-dependences of 2 μM ≡-CLICK-17 and ≡-PERMUT-17 in the presence of 5 μM and 20 μM Cu^+^, respectively. The error bars shown represent one standard deviation from the mean, determined from three independent experiments.

Detailed time-dependence measurements of *in cis* CuAAC (biotinylation) experiments were carried out for the first 100 min of reaction, for both 2 μM ≡-CLICK-17 and 2 μM ≡-PERMUT-17, at different Cu^+^ concentrations (Supporting Figures S4 and S5). Supporting Figure S4 shows that in the presence of as little as 5 μM Cu^+^, the ≡-CLICK-17 reaction is 80% complete within 20 min; with 8 nM Cu^+^, >20% conversion occurs in 2 h. A striking observation about the Cu^+^-dependence of the ≡-CLICK-17 reaction is that highest catalytic activity is observed with ∼ 5 μM Cu^+^. Reaction with 20 μM Cu^+^ is dramatically slower (slower than with 1 μM or 5 μM Cu^+^). The most plausible explanation is that at ∼20 μM Cu^+^, ≡-CLICK-17 forms an alternatively folded conformer that is either non-catalytic or poorly catalytic. Indeed, Figure 3, which plots *in cis* biotinylation rates for ≡-CLICK-17 and for ≡-PERMUT-17 for the first 16 min of reaction, shows that at 5 μM Cu^+^, the ≡-CLICK-17 reaction is ∼2000-fold more rapid than the ≡-PERMUT-17 reaction. However, at 20 μM Cu^+^, the ≡-CLICK-17 and ≡-PERMUT-17 rates are comparable, likely representing baseline CuAAC rates observable with 2 μM of *any* 79-nt long ≡-ssDNA.

Supporting Figure S6 shows ESI mass spectrometry data that confirm that the streptavidin-retarded products on gels, such as shown in Figure 2A, are indeed the CuAAC adduct of ≡-CLICK-17 and azide-biotin. To facilitate ESI mass spectrometry of the conjugation product, the CuAAC-reacted mixture was subjected to complete digestion with Nuclease P1, an endonuclease that cleaves DNA into 5′-deoxyribonucleotide monophosphates. The resulting residual structure from the expected conjugation product of ≡-CLICK-17 with azide-biotin incoporates the 5′-most deoxyriboguanosine monophosphate residue of the CLICK-17 DNA sequence linked to biotin via the triazole and linker atoms shown in Supporting Figure S6.

### The *in trans* reaction: CLICK-17 is a true enzyme for CuAAC

Given that ≡-CLICK-17 is capable of harnessing very low (≤5 μM) concentrations of Cu^+^ to efficiently catalyze the CuAAC reaction *in cis*, two questions arose immediately: (a) what mechanistic role does the CLICK-17 DNA play? A ‘minimal’ hypothesis would be that CLICK-17 plays a role similar to Cu^+^-stabilizing ligands such as THPTA. (b) Can CLICK-17 DNA (now *underivatized* with the hexynyl moiety) catalyze CuAAC *in trans*, utilizing *both* diffusible alkyne and azide substrates?

To investigate whether folded CLICK-17 DNA can catalyze CuAAC *in trans*, we first explored suitable assays for making such a determination. We picked 5-hexyn-1-ol (‘hexynol’) as a diffusible alkyne substrate on the basis of its similarity to the 5-hexynyl moiety within ≡-CLICK-17. However, azide-biotin here was not a particularly useful substrate, because its expected triazole product with hexynol would be a low molecular weight compound requiring specialized detection procedures. We therefore examined a structurally distinct, fluorogenic azide (3-azido-7-hydroxycoumarin or ‘azide-coumarin’) as a potential substrate for CLICK-17. Experiments were carried out in R buffer with 2 μM CLICK-17 DNA, 8 mM hexynol and 50 μM (limiting) azide-coumarin. The progress of any reaction could be followed using fluorescence measurements (excitation at 404 nm and emission at 480 nm). A fluorescence calibration curve, quantitatively linking measured fluorescence to triazole product concentration, is shown in Supporting Figure S7.

Supporting Figure S8 shows that both azide-coumarin and hexynol were diffusible substrates acceptable to CLICK-17 for CuAAC catalysis *in trans*. The initial rate for the CLICK-17-catalyzed reaction with 5 μM Cu^+^ was 0.24 μM min^−1^; that for PERMUT-17 DNA was < 0.01 μM min^−1^; that in the absence of all DNA was 0.03 μM min^−1^. We compared CuAAC kinetics measured with a fixed Cu^+^ concentration (5 μM) in the presence of: 2 μM CLICK-17 and no THPTA; 25 μM THPTA and no DNA; neither DNA nor THPTA; as well as with both DNA and THPTA present together. Supporting Figure S8 shows that 2 μM CLICK-17 is ∼1.2-fold more efficient as a catalyst than 25 μM THPTA under these conditions. Interestingly, CLICK-17 and THPTA together do not show any synergy. Moreover, the complete lack of catalysis observed with 2 μM of the control PERMUT-17 DNA cannot be rescued by the presence of THPTA.

The above results suggest that for its catalytic action using Cu^+^, CLICK-17 likely provides privileged binding site(s) as well as significant redox stabilization for one or more catalytically important Cu^+^ ion(s) within its active site. As the data on the non-catalytic PERMUT-17 DNA demonstrate, beyond such privileged binding, DNA sequesters and renders CuAAC-unavailable any residual Cu^+^ in the solution under these reaction conditions. Not only does PERMUT-17 DNA kill CuAAC comprehensively; in addition, its likely non-specific sequestration of copper cannot be reversed or rescued by THPTA.

To optimize *in trans* catalysis by CLICK-17, we explored whether the inhibition imposed by higher (20 μM) Cu^+^ on the activity of 2 μM CLICK-17 could be overcome by varying the CLICK-17 concentration in the fixed presence of 20 μM Cu^+^. Supporting Figure S9 shows that, indeed, CuAAC levels obtained with 4 μM CLICK-17 are >3-fold higher than those obtained with 2 μM CLICK-17. The complex dependence of CuAAC rates as a function of CLICK-17 concentration (at fixed [Cu^+^]) likely results from the distinct modes of Cu^+^ binding to CLICK-17, i.e. tight binding to the active site as well as non-specific binding along the length of the DNA.

### CLICK-17 is catalytic with added Cu^2+^ in the absence of explicit reductants

The most striking observation from the *in cis* experiments with ≡-CLICK-17 (Figure 2A) was that in the presence of 100 μM CuSO_4_, with no ascorbate (or other extrinsic reducing agent) added, CuAAC was still catalyzed to a high level. By contrast, under those same conditions, no *in cis* reaction was seen with the control alkynyl DNA, ≡-PERMUT-17 (Figure 2B).

To further understand these observations, a thorough investigation was carried out with (unalkynated) CLICK-17 DNA, first, under *in trans* conditions.

**Figure 4.**
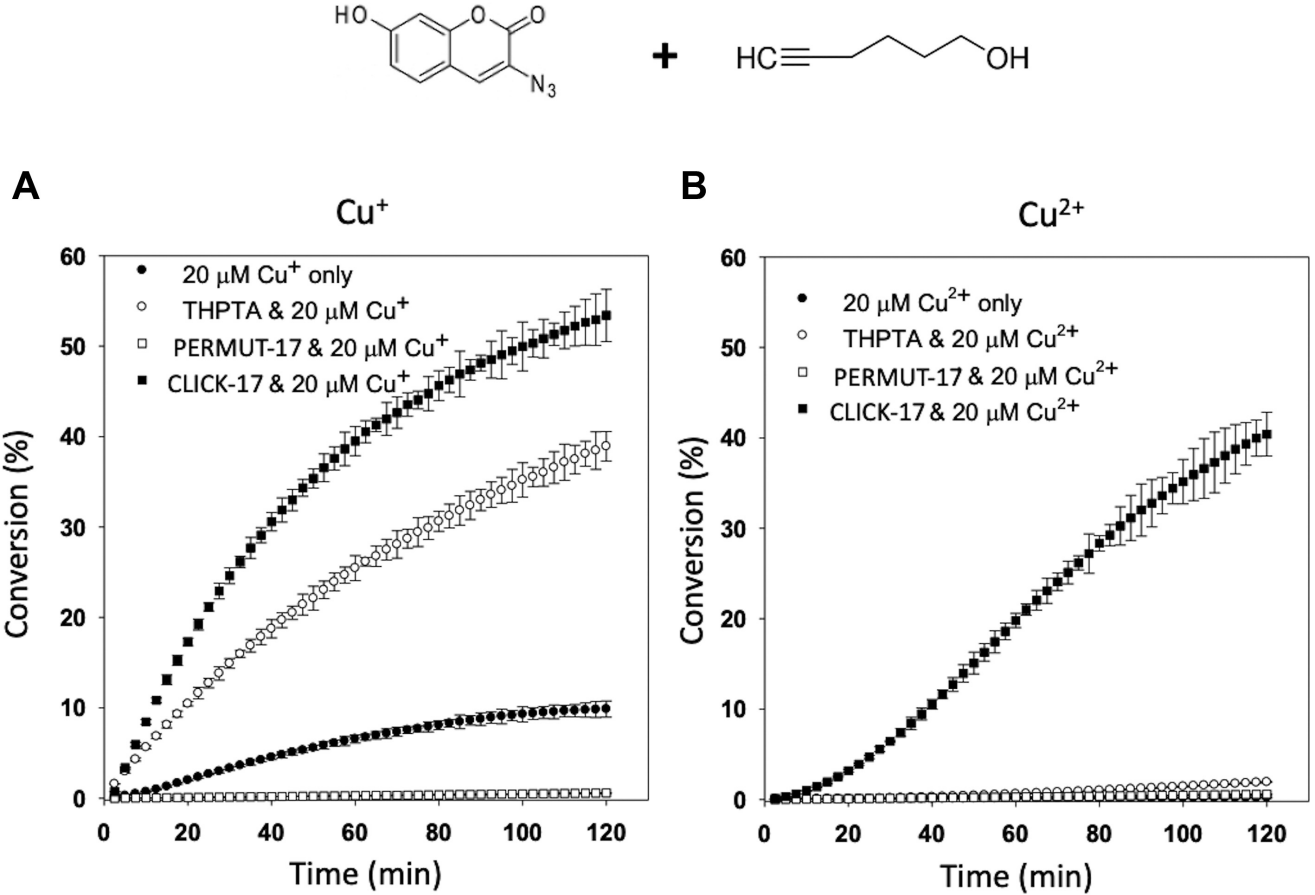
CLICK-17 also catalyzes the *in trans* reaction with either added Cu^+^ or Cu^2+^. The reactions were carried out in R buffer (50 mM Li-HEPES, pH 7.4, 20 mM MgCl_2_) with 4 μM DNA (CLICK-17 or PERMUT-17). Panels A and B show time-courses of the CuAAC reaction between hexynol and azide-coumarin catalyzed by CLICK-17 DNA, compared to PERMUT-17 DNA, THPTA, or in the absence altogether of added ligand or DNA. Concentrations of the fluorescent triazole product are shown as percentages of the starting concentration (50 μM) of the limiting substrate, azide-coumarin. Whereas the kinetic curves in the presence of added Cu^+^ are hyperbolic (panel **A**), in the presence of Cu^2+^, the CLICK-17-catalyzed time-dependence is sigmoidal in shape (panel **B**). The error bars shown represent one standard deviation from the mean, determined from three independent experiments.

Figure 4A and B shows side-by-side comparisons of CuAAC catalyzed in the presence of 20 μM Cu^+^ (generated *in situ* by 2.5 mM ascorbate—panel (A); and, in the presence of 20 μM Cu^2+^ in the absence of ascorbate (panel B). 4 μM of either CLICK-17 or PERMUT-17 DNA (at 4 μM, CLICK-17 is optimally active with 20 μM Cu^+^: Supporting Figure S9) was used, with hexynol and azide-coumarin as diffusible substrates. Panel A shows that (a) Cu^+^ in the absence of either THPTA or CLICK-17 DNA was poor at promoting CuAAC (10% conversion after 2 h); (b) either 100 μM THPTA (∼40% conversion) or 4 μM CLICK-17 (∼55% conversion) accelerated CuAAC significantly and (c) the presence of 4 μM of the control PERMUT-17 DNA abrogated the reaction completely.

The Cu^2+^ data shown in Figure 4B, however, were strikingly different. With 20 μM CuSO_4_ in the reaction (in the absence of added ascorbate), *only* CLICK-17 was able to catalyze the reaction (∼40% conversion after 2 h). All the other controls (Cu^2+^ alone; Cu^2+^ plus THPTA; and Cu^2+^ plus PERMUT-17 DNA) failed to support the reaction.

A striking feature of the time-dependence of the CLICK-17/Cu^2+^ combination is its *sigmoidal* rather than hyperbolic shape, indicative of an initial ‘lag’ or ‘induction’ phase. Such kinetics are consistent with the need to reduce Cu^2+^ and the *in situ* generation of a sufficient concentration of catalytically obligatory Cu^+^, stabilized by CLICK-17, to promote CuAAC (a comparison of the relative efficacies of added Cu^+^ versus Cu^2+^ under optimized reaction conditions, where CuAAC proceeds to completion in 30–40 min, is shown in Supporting Figure S10).

### *In trans-* and *in cis*- labeling of macromolecules (proteins) using the CLICK-17 DNAzyme

We wished to explore the utility of the CLICK-17 DNAzyme (i) to catalyze the conjugation of an azido-protein *in trans* with an alkynated fluorescent dye; as well as (ii) to catalyze the *in cis* conjugation of a fluorescently-labeled version of itself (≡-CLICK-17-Fluorescein) to the azido-protein. We wished to test for these activities under the very low Cu^+^ and Cu^2+^ concentration regimes that were shown, above, to be characteristic of CLICK-17’s activity with small molecule substrates.

First, we labeled the protein lysozyme (14,305 Da) with Azido-PEG2-NHS Ester. The purified products were analyzed by LC–mass spectrometry (ESI). Supporting Figure S11 shows a near-complete conversion of the unlabeled lysozyme to three new products of MW 14,489, 14,674 and 14,859 Da, corresponding to derivatized lysozymes with one, two, and three appended azide functionalities, respectively (each label adds ∼185 Da to the protein). This mixture of azido-lysozymes (hereafter referred to as lysozyme-(N_3_)_1–3_) was then tested first, for *in trans* coupling with alkyne labeled Alexa Fluor Dye 546 (≡-AFDye 546).

**Figure 5.**
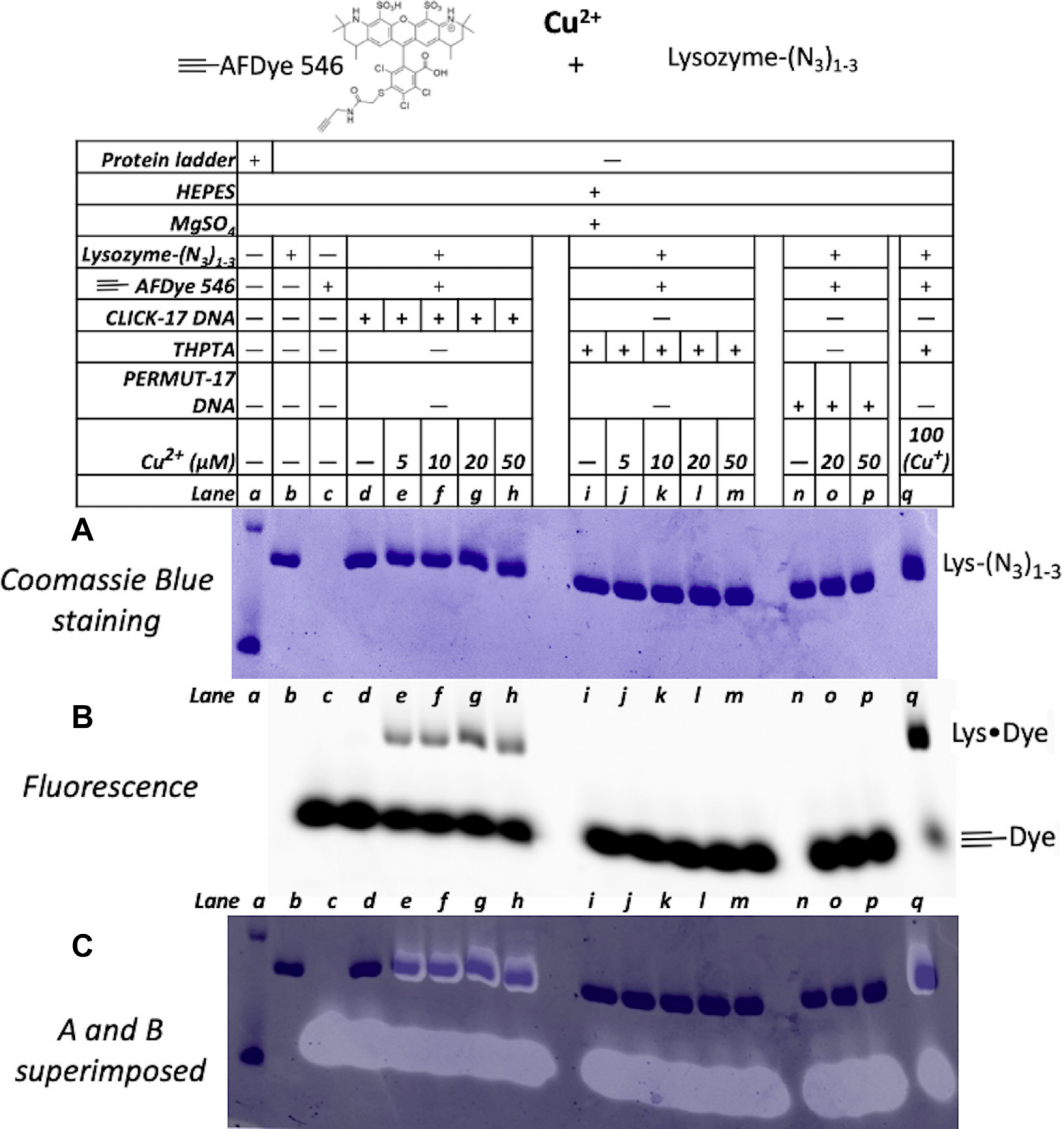
SDS-PAGE gel showing Cu^2+^-dependence of *in trans*-catalyzed coupling by CLICK-17 of alkynated Alexa Fluor 546 dye (≡-AFDye 546) to an azide-labeled protein (lysozyme-(N_3_)_1–3_). Coomassie Blue-staining patterns for protein in (**A**); AFDye 546 fluorescence is shown in (**B**); and overlap of the two gels is shown in (**C**). Lanes, from left to right, show a protein ladder (the reference bands seen in this lane in panels (B) and (C) represent 10 and 15 kDa standards); lysozyme-(N_3_)_1-3_ only; ≡-AFDye 546 only; lysozyme-(N_3_)_1-3_ incubated for 2 h with ≡-AFDye 546 in the absence of added copper; the two reactants incubated for 2 h in the presence of 5 μM Cu^2+^; 10 μM Cu^2+^; 20 μM Cu^2+^; and, a positive control of the two reactants with 0.1 mM Cu^+^/0.5 mM THPTA for 2 h.

Figure 5 shows an SDS PAGE gel with the results of Cu^2+^ and CLICK-17-catalyzed *in trans* coupling of 4 μM lysozyme-(N_3_)_1–3_ with 20 μM ≡-AFDye 546, in R buffer for 2.5 h, at 22°C. Panel A shows Coomassie Blue-labeled protein bands, while panel B shows the fluorescence of AFDye 546. The second lane from the left in the gel was loaded with ≡-AFDye 546 alone. Panel B therefore identifies the lower of the two fluorescent bands in the gel as free ≡-AFDye 546 (‘≡-Dye’). The upper fluorescent band, generated with 5–50 μM Cu^2+^ in the presence of CLICK-17 (but *not* with PERMUT-17 *nor* a 5-fold molar excess of THPTA), was therefore hypothesized to represent the protein–dye conjugate. Indeed, panel C, which overlaps the protein mobility data of panel A with the dye fluorescence data of panel B, shows that this hypothesis is correct. The gel band to the extreme right in all panels shows a positive control, CuAAC under standard conditions used in the field (100 μM Cu^+^ in the presence of 500 μM THPTA). Although the yield of lysozyme-dye conjugate catalyzed by CLICK-17/Cu^2+^ under these conditions is ∼40% of that achieved with the positive control, it must be emphasized that the CLICK-17 here is using Cu^2+^ as its *exclusive cofactor* (in the absence of any explicit reductant). In the presence of Cu^+^, CLICK-17 performs comparably to the positive control. LC–MS analysis of the dye-lysozyme CuAAC conjugates obtained, as shown in Figure 5, are given in Supporting Figures S12 and S13.

**Figure 6.**
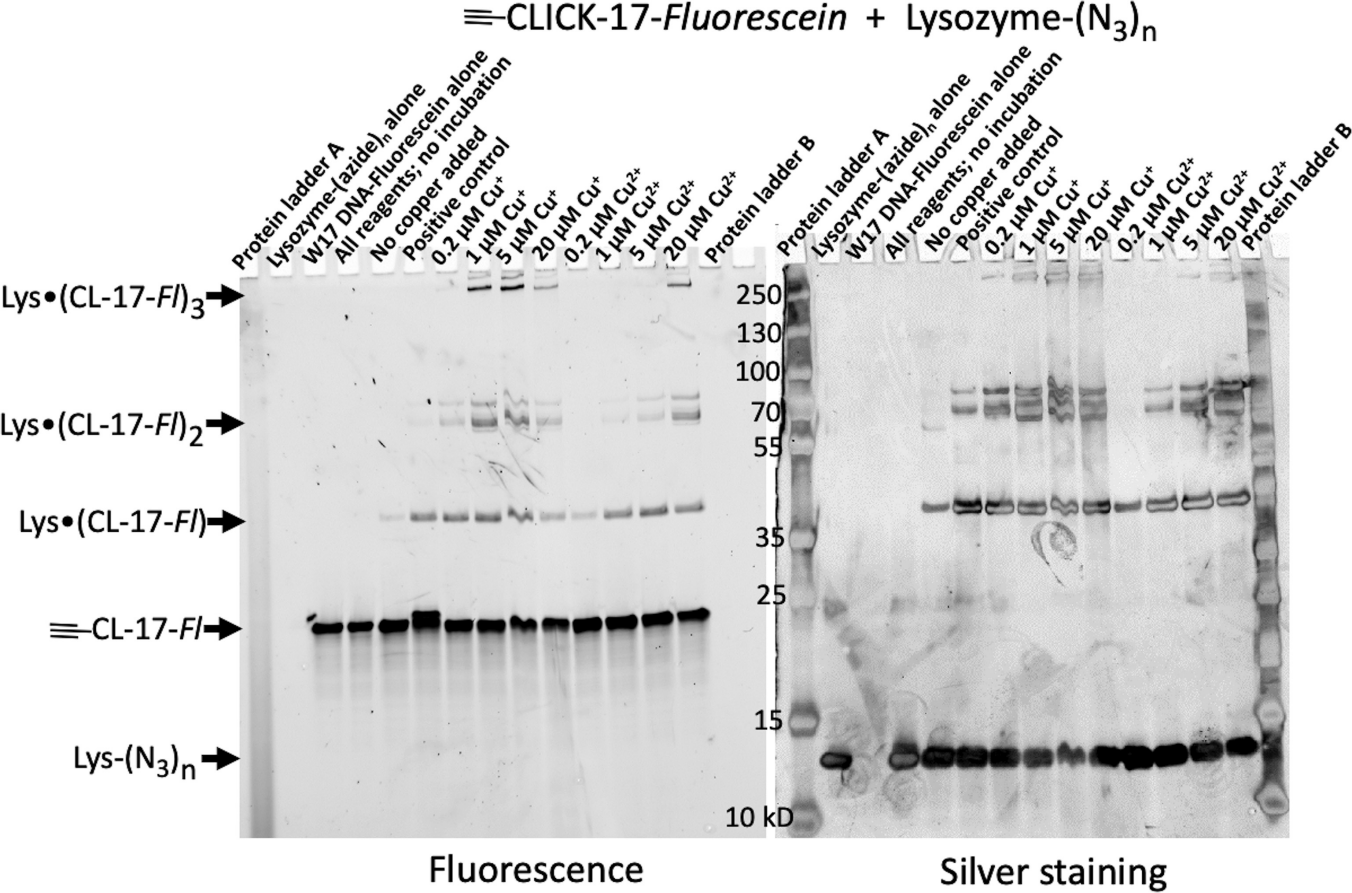
SDS-PAGE gel showing Cu^+^- and Cu^2+^-dependence of *in cis*-catalyzed clicking of fluoresceinated ≡-CLICK-17 DNA to an azide-labeled protein (lysozyme-(N_3_)_1–3_). Both fluorescein fluorescence, indicative of DNA (*left*) and silver staining patterns for protein (*right*) are shown, both in grey scale. Lanes, from left to right, show Protein Ladder A; lysozyme-(N_3_)_1–3_ only; ≡-CLICK-17-Fluorescein only; lysozyme-(N_3_)_1–3_ mixed with ≡-CLICK-17-Fluorescein in the absence of either added copper or incubation; lysozyme-(N_3_)_1–3_ and ≡-CLICK-17-Fluorescein without added copper but incubated for 2.5 h; a positive control of the two reactants with 1 mM Cu^+^/5 mM THPTA for 2.5 h; the two reactants incubated for 2 h in the presence of 0.2 μM Cu^+^; 1 μM Cu^+^; 5 μM Cu^+^; 20 μM Cu^+^; 0.2 μM Cu^2+^; 1 μM Cu^2+^; 5 μM Cu^2+^; 20 μM Cu^2+^; and, Protein Ladder B.

Figure 6 shows the results of the ≡-CLICK-17-catalyzed *in cis* conjugation of lysozyme-(N_3_)_1-3_ to the fluorescein-labeled ≡-CLICK-17 DNAzyme itself. The SDS PAGE gel shows the results of incubation of 1 μM lysozyme-(N_3_)_1–3_ and 2 μM ≡-CLICK-17-Fluorescein DNA, in R buffer for 2.5 h at 22°C. Both fluorescein fluorescence (from DNA—Figure 6, *left*) and protein silver staining (rather than Coomassie staining, because of the very low protein concentrations used) patterns (Figure 6, *right*) are shown. The positive control experiment here is the reaction of 2 μM ≡-CLICK-17-Fluorescein with 1 μM lysozyme-(N_3_)_1-3_ under ‘normal’ CuAAC conditions (i.e. with 1 mM Cu^+^ and 5 mM THPTA). The key observation in Figure 6 is the appearance of three sets of product bands, visible with both fluorescence and silver staining detection (i.e. these products contain *both* DNA and protein, hypothesized to be the 1:1, 1:2 and 1:3 protein: DNA adducts; confirmatory mass spectra are shown in Supporting Figures S14 and S15). These products are labeled, accordingly, as ‘Lys•(CL-17-Fl)’, ‘Lys•(CL-17-Fl)_2_’ and ‘Lys•(CL-17-Fl)_3_’. There is a single band associated with ‘Lys•(CL-17-Fl)’, consistent with it showing a 1:1 protein–DNA conjugate; two or more closely spaced bands can be seen for ‘Lys•(CL-17-Fl)_2_’ (and for ‘Lys•(CL-17-Fl)_3_’), likely representing protein-DNA conjugates of the same molecular weight but with variable sites of attachment (and hence, diverse geometries and gel mobilities) of two or three ≡-CLICK-17 DNAs conjucated to one lysozyme-(N_3_)_1–3_ molecule.

What the Figure 6 gel strikingly shows—seen particularly clearly from the DNA fluorescence bands on the *left*—is that (i) under our experimental conditions, as little as 0.2 μM Cu^+^ or 1 μM Cu^2+^ catalyze formation of the 1:1 ≡-CLICK-17-lysozyme conjugate (‘Lys•(CL-17-Fl)’) comparably as well as the positive control (under optimal CuAAC conditions: 1 mM Cu^+^/5 mM THPTA). (ii) Somewhat higher Cu^+^ (1 μM) or Cu^2+^ (5 μM) catalyze the formation of the 2:1 and 3:1 ≡-CLICK-17-lysozyme conjugates at *significantly higher levels* than does the positive control.

As seen earlier, with ≡-CLICK-17 *cis-*catalyzed small molecule conjugations (Figure 2), even in incubations wholly lacking copper, ≡-CLICK-17 does promote a low level of DNA-protein conjugate-formation—consistent, again, with the existence of an active site within folded CLICK-17/≡-CLICK-17. Given that CLICK-17 is a macromolecular catalyst, with a catalytically relevant folded structure, we see in Figure 6 (as also in Figures 2 and 3) that higher concentrations of Cu^+^ (for example, 20 μM in Figure 6) disfavor CLICK-17’s catalysis. Again, this is because at such higher [Cu^+^], CLICK-17 forms an alternative, less catalytic or non-catalytic fold.

We carried out ESI mass spectrometry analysis on the mixture of species formed in the above *in cis* catalyzed conjugation of ≡-CLICK-17 DNA with lysozyme-(N_3_)_1-3_. Supporting Figure S14, panels A and B, show, respectively, schematic diagrams of unmodified lysozyme and lysozyme-(N_3_)_*n*_ (where *n* = 1). Panel C shows the expected *in cis* conjugated product of lysozyme-(N_3_)_1_ reacted with ≡-CLICK-17 (the full length of the conjugated CLICK-17 is shown as ‘DNA’). To facilitate ESI mass spectrometry identification of this latter product, the DNA component of the protein–DNA conjugate was digested to completion with Nuclease P1, an endonuclease that cleaves DNA into 5′-deoxyribonucleotide monophosphates. The portion of the appended CLICK-17 DNA expected to be removed by the nuclease is indicated by a red bracket. The resulting residual structure incoporates the 5′-most deoxyriboguanosine residue of the CLICK-17 sequence conjugated to the lysozyme via the triazole and linker atoms, as indicated. Panel D provides the schematics of symbols used for the ESI mass spectrometry data for conjugation of *in cis* catalyzed conjugation of ≡-CLICK-17 DNA with lysozyme-(N_3_)_1–3_ shown in Supporting Figure S15. In this last figure, in addition to low amounts of the starting materials, underivatized lysozyme (‘A’), lysozyme-(N_3_) (‘B’), lysozyme-(N_3_)_2_ (‘C’) and lysozyme-(N_3_)_3_ (‘D’), bands corresponding to five DNA conjugated (following nuclease digestion) species, ‘E’ to ‘I’, can be seen, by far the most abundant of which is ‘F’ [lysozyme-(N_3_)_2_•(CLICK-17 digestion residue)_1_].

### Specific nucleotide sequences and foldings are required for CLICK-17 and CLICK-16 catalytic activity

To further confirm that correct folding of CLICK-17 DNA is a key factor for its catalytic competence, we examined the catalytic activity of two other DNA clones from our original SELEX experiment, CLICK-16 and CLICK-20, both with sequences closely related to that of CLICK-17. Second, we tested a number of rationally designed mutants derived from CLICK-17, CLICK-16, and CLICK-20. Third, we subjected the nucleotide sequences of CLICK-17, CLICK-16 and CLICK-20 to the folding algorithm, Mfold, to try and correlate catalytic activity with their predicted folded structures (31).

Supporting Figure S16, *upper*, shows the nucleotide sequences of the SELEX-derived DNA clones CLICK-17, CLICK-16 and CLICK-20, as well of the three mutants, CLICK-17_T24G, CLICK-20_C39G and CLICK-20_G24T. 77-nucleotide long CLICK-16 differs from 79-nt CLICK-17 in a single point mutation and in lacking two adjacent thymines present in CLICK-17. 79-nt CLICK-20 varies from CLICK-17 in having three non-adjacent point mutations. The three further sequences tested restore some of the nucleotide differences between CLICK-17, CLICK-16 and CLICK-20, listed above. Supporting Figure S16, *lower*, shows that these oligonucleotides are notably different in their ability to utilize either Cu^+^ or Cu^2+^ to catalyze CuAAC *in trans*. Specifically, CLICK-16 is catalytic, though less so than CLICK-17; while CLICK-20, CLICK-20_C39G and CLICK-20_G24T are inactive under these reaction conditions. These data further emphasize that CLICK-17’s catalytic properties are precisely dependent on its nucleotide sequence and its concomitantly folded structure. The nucleotide sequences of the catalytic CLICK-17 and CLICK-16 as well as the non-catalytic CLICK-20 were subjected to the Mfold program used for predicting the most stably folded structures of single-stranded DNAs (31). Supporting Figure S17 shows the thermodynamically most stable predicted folds for the three DNAs. It is interesting to note that the catalytically active CLICK-17 and CLICK-16 share a common fold, whereas the inactive CLICK-20 shows a divergent folded structure.

### Investigations into the mode of action of CLICK-17

The data shown above cumulatively encourage a hypothesis that CLICK-17 is a DNA enzyme with a precise, catalytically active fold, and an active site that offers one or more privileged bindings sites for Cu^2+^ or Cu^+^. It is hypothesized that such a binding site/sites powerfully impacts the redox potential of Cu^2+^/Cu^+^ in a positive direction, i.e. toward Cu^+^. Therefore, CLICK-17 shows catalytic activity (both *in cis* and *in trans*) with added Cu^2+^ alone, in the absence of any explicit reductant such as ascorbate. Conceivably, one or more Cu^2+^ ion/ions bound to the active site are then relatively easily reduced to the CuAAC-capable Cu^+^ species not by a classic reductant such as ascorbate, but an available buffer solution component, such as HEPES.

**Figure 7.**
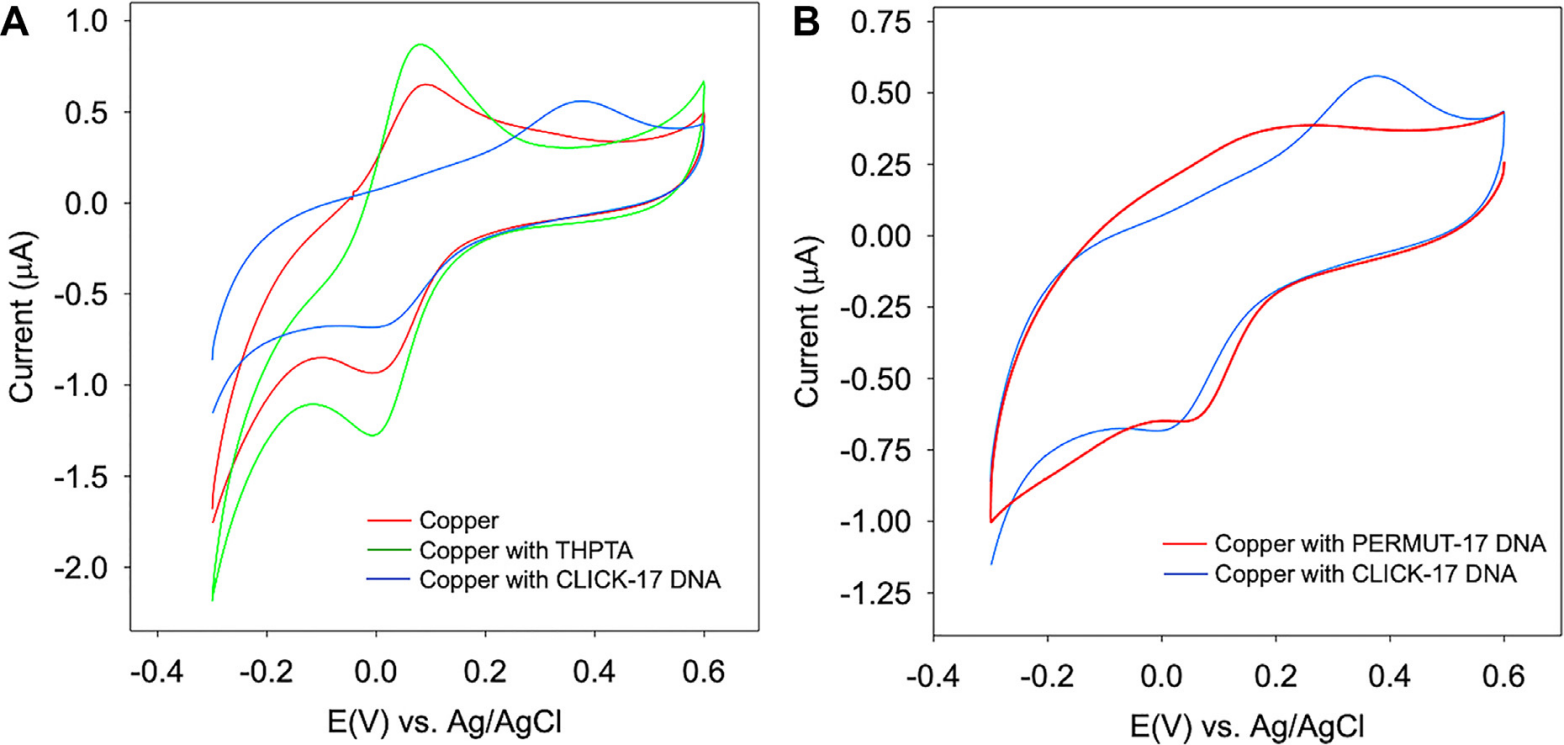
Cyclic voltammetry of Cu^2+^/Cu^+^ with different ligands. (**A**) cyclic voltammetry of 0.1 mM CuSO_4_ in HEPES buffer (red); 0.1 mM CuSO_4_ with 0.5 mM THPTA in HEPES buffer (green); 0.2 mM CuSO_4_ with folded 0.1 mM CLICK-17 DNA (blue). (**B**) 0.2 mM CuSO_4_ with folded 0.1 mM CLICK-17 DNA (blue); and 0.2 mM CuSO_4_ with folded 0.1 mM PERMUT-17 DNA (red).

To test, first, the redox potential of copper ion bound to CLICK-17, we carried out cyclic voltammtry (CV) experiments. Figure 7 shows the results. The presence of the DNAzyme, CLICK-17, significantly influences the redox property of the Cu(II)/Cu(I) couple in the HEPES buffer. While there are no discernible changes in either the reduction or the oxidation peak with added THPTA, the oxidation peak appears 301 mV more positive when CLICK-17 is introduced to the electrolyte. This corresponds to a ∼150 mV shift in the formation potential of Cu(II)/(I), indicative of the much improved stability of Cu(I). In addition, the much larger separation between the oxidation and reduction peaks indicates slower electron-transfer rates between the two forms as well. In comparison, the presence of the folded, uncatalytic DNA strand (PERMUT-17) induces no such substantial changes to the CV, i.e., the formal potential shift is not as significant; and, the oxidation peak becomes broad. The above observations are consistent with the binding of Cu(I)/Cu(II) species to the CLICK-17 DNAzyme as being both strong and unique.

An examination of the extant literature for other reported instances of CuAAC observed in the presence of Cu^2+^ (in the absence of extrinsic reducing reagents) revealed a small number of reports of such events (32–42). Unsuspected reductants were, however, ultimately identified in those systems—most notably, excess alkyne, capable of reducing Cu(II) to Cu(I) via alkyne homocoupling (33–35); alternatively, a role for alcohol oxidation has also been proposed (33).

**Figure 8.**
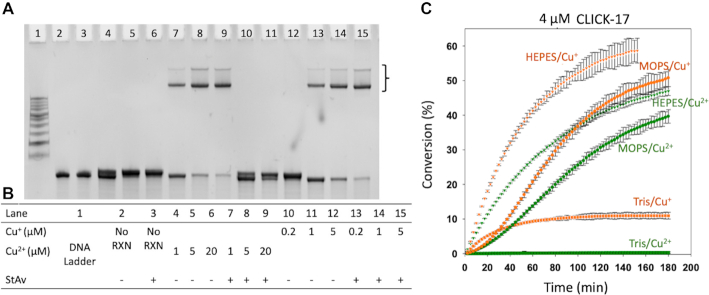
Investigation of Cu^2+^-induced alkyne homocoupling and the role of buffer components in the generation of Cu^+^ for CuAAC by CLICK-17 and ≡-CLICK-17. (**A**) Investigation of alkyne homocoupling using the *in cis* reaction of ≡-CLICK-17 with azide-biotin. The bracket indicates streptavidin-shifted bands of biotinylated ≡-CLICK-17. The DNA band running just above the unreacted ≡-CLICK-17 represents the triazole product formed between ≡-CLICK-17 and azide-streptavidin. However, no additional DNA band corresponding to a CLICK-17-≡-≡-CLICK-17 product can be seen in any of the lanes. (**B**) Table of reagents used for the data shown in (A). (C) Investigation of the effectiveness of different buffering agents in promoting the *in trans* catalysis of conjugation between hexynol and azide-coumarin, catalyzed by CLICK-17, in the presence of either Cu^+^ or Cu^2+^. The error bars represent one standard deviation from the mean obtained from three independent experiments.

To investigate whether alkyne homocoupling might be responsible for our observed CLICK-17/‘Cu^2+^’ catalysis, a close investigation of the *in cis* reaction of ≡-CLICK-17 was made. In the *in cis* reaction, the only alkynes present are the hexynyl moieties covalently appended to CLICK-17 DNA. If alkyne homocoupling were indeed occurring, two ≡-CLICK-17 strands would be joined end-to-end to yield a product (‘CLICK-17-≡-≡-CLICK-17’) of twice the length and molecular weight of 2 x CLICK-17 (strictly, a molecular weight of [(2 x CLICK-17) -2]), which should show a predictable electrophoretic mobility that should be observable in a denaturing polyacrylamide gel. Figure 8, panel A, shows that in the presence of either Cu^2+^ or Cu^+^, *only* streptavidin retarded bands but *no additional DNA band* corresponding to a 158-nt DNA product is present in the gel (lanes 4–6). DNA bands of very slightly higher molecular weight, running just above the unmodified ≡-CLICK-17 bands, are discernable with *both* Cu^2+^ (lanes 4–6) and Cu^+^ (lanes 10–12)—this is the triazole product formed by ≡-CLICK-17 with azide-biotin.

We have shown that binding to CLICK-17 DNA (but not to PERMUT-17 DNA) dramatically shifts the redox potential of Cu^2+^/Cu^+^ towards Cu^+^. There nevertheless remains the requirement of an electron source for the reduction. It is clear that a powerful reductant such as ascorbate is not required by CLICK-17/Cu^2+^. Figure 8, panel ***b***, shows the effectiveness of different buffer solutions in promoting CLICK-17-catalyzed *in trans* CuAAC by Cu^+^ as well as by Cu^2+^. It is evident that while the Cu^+^ reaction works in all three buffers tested (albeit less well in Tris (8)), the CLICK-17/Cu^2+^ catalysis is promoted by HEPES and MOPS but not at all by Tris. It has been reported that HEPES can act as a mild reductant (43)—thus, it is plausible that it acts as the mild reductant necessary to convert sufficient CLICK-17-bound Cu^2+^ to the rquired CuAAC-active Cu^+^.

To address a potential role for alcohol oxidation (33) in this catalytic system, a number of our substrates were indeed alcohols (hexynol, propargyl alcohol). Nevertheless, deliberate supplementations of the *in trans* reaction with exogenous methanol (5% and 15%, v/v, respectively) led to *less* effective CuAAC rather than to rate acceleration (Supporting Figure S16, panel A). Crucially, the sigmoidal shapes of the rate profiles (and unchanging lag-times of 20–30 min) persisted irrespective of the alcohol content of these reactions, discouraging the hypothesis of a determining role for alcohol oxidation in CLICK-17’s Cu^2+^ reaction.

We also tested for whether any residual azide anion or a contamination of amines in the commercially synthesized azide substrates that we have used, could act as reductants for CLICK-17’s ‘Cu^2+^’ reaction. Supporting Figure S18 shows that supplementations of up to 20 μM of either the N_3_^−^ anion or of methylamine did not change the catalytic rate profiles of CLICK-17, nor did they change the characteristic sigmoidal shapes of these profiles.

Given that CLICK-17 is catalytic both *in cis* (as ≡-CLICK-17) and *in trans* and is able to accept structurally divergent azide substrates (azide-biotin, azide-coumarin, and azide-labeled lysozyme) and alkynes (hexynol, propargyl alcohol, Alexa Fluor Dye 546), we attempted to determine a putative *K*_M_ value for one of our substrates, hexynol. Supporting Figure S19 shows the reaction profiles of Cu^+^/CLICK-17-catalyzed CuAAC with a fixed concentration (50 μM) of azide-coumarin and varying concentrations (0.1–20 mM) of hexynol. It was concluded that the *K*_M_ for hexynol with respect to CLICK-17 was >20 mM.

## DISCUSSION AND FUTURE PROSPECTS

The data reported herein demonstrate that the 79-nt DNA oligonucleotide, CLICK-17, is a *bona fide* enzyme for the copper-dependent azide-alkyne cycloaddition (CuAAC) reaction. Folded CLICK-17 is a metalloenzyme, able of harness ascorbate-generated Cu^+^, as well as added Cu^2+^ in the absence of ascorbate, with buffer components as likely reductants for CLICK-17’s catalyze CuAAC. Curiously, guanine bases in DNA and RNA are relatively easily oxidized and are ready sources of electrons for such phenomena as the recently discovered intrinsic photoreactivation of cyclobutane thymine dimers within DNA (44–47). It is therefore conceivable that one or more guanines bases within CLICK-17’s sequence may also contribute to the Cu^2+^ to Cu^+^ reduction. This will be the subject of further investigation.

We show, using cyclic voltammetry, that complexation of copper ions to CLICK-17 DNA (but not to a sequence-permuted DNA isomer, PERMUT-17) perturbs the Cu(II)/Cu(I) redox potential, by approximately 150 mV relative to that of copper ions in the presence of either HEPES buffer alone or containing THPTA. These data are consistent with folded CLICK-17 DNA (but not folded PERMUT-17 DNA) providing one or more high-affinity and catalytically relevant copper ion binding sites within CLICK-17’s active site.

CLICK-17 is CuAAC-active with Cu^2+^ in reaction solutions buffered by the mildly reducing HEPES and MOPS but not by Tris. Likely, HEPES and MOPS are sufficiently reducing for the CLICK-17’s bound (and redox-potential shifted Cu^2+^ ion/ions—see above) but not for Cu^2+^ bound to PERMUT-17 or to THPTA. Above, we also show that neither alkyne homocoupling nor adventitious reduction of Cu^2+^ to Cu^+^ by primary alcohols—two alternative mechanisms invoked for Cu^2+^-dependent CuAAC observed in other experimental systems that have been described in the literature, is responsible for the observed Cu^2+^-dependent activity of CLICK-17.

We do not yet know any details of the ligand environment or environments provided by CLICK-17. CLICK-17 has 79 heterocyclic nucleobases (of which 23 are guanines); and, at pH 7.4, 79 full negative charges. Several studies have examined the preferred copper-binding sites in DNA. Sigel and Sigel (29) have described that the guanine N7 and O6 positions, together with a negatively charged phosphate oxygen, provides a tight multidentate binding site for Cu^2+^ (with estimated formation constant of ∼10^4^ M^−1^). The binding of Cu^+^ to DNA may be stronger yet; association constants of ∼ 10^9^ M^−1^ have been reported (28). A viable cytosine-Cu^+^-cytosine complex has also been reported in DNA (30). However, the ligand environment in CLICK-17’s active site for binding one or more Cu^2+^/Cu^+^ ions may be yet more intricate, conceivably involving Cu^+^/Cu^+^ or Cu^2+^/Cu^+^ bimetallic binding sites such as have been observed elsewhere (34).

Future work will focus on generating and studying truncated pieces of CLICK-17 that may be still capable of efficiently catalyzing CuAAC with both Cu^+^ and added Cu^2+^. These, and other studies will help us to fully understand the mechanism of CLICK-17.

We have shown that CLICK-17 itself, though it is a prototypical CuAAC enzyme, is not especially substrate-specific, and will accept a variety of small molecule as well as macromolecular azide (azide-biotin; azide-coumarin; azide-labeled lysozyme) as well as alkyne (≡-CLICK-17; hexynol; propargyl alcohol) substrates. This substrate *generality* is an unexpected and peculiar virtue of CLICK-17. However, we do expect to use SELEX to obtain Cu^2+^-dependent, CuAAC-catalyzing DNA enzymes that are highly *substrate specific*. A well-established variant of SELEX, called ‘counter-SELEX’ has widely been used (48,49) to select for DNAzymes and ribozymes that show high substrate-specificity. Indeed, we have earlier used counter-SELEX with great success for obtaining ribozymes that utilize thiamin as a cofactor for decarboxylating α-keto acid substrates (50).

CLICK-17, as a prototypical enzyme for the CuAAC ‘click’ reaction, is both larger in size (79 nt; ∼26 kD) and more expensive than a small molecule ligand such as THPTA (∼0.5 kD). However, automated DNA synthesis in recent years has become both highly efficient and low-cost; so that the cost differential between the two is likely not as large as may be imagined. Over and above size and cost issues, CLICK-17 and other DNA enzymes are likely to offer the following unique advantages.

**Figure 9.**
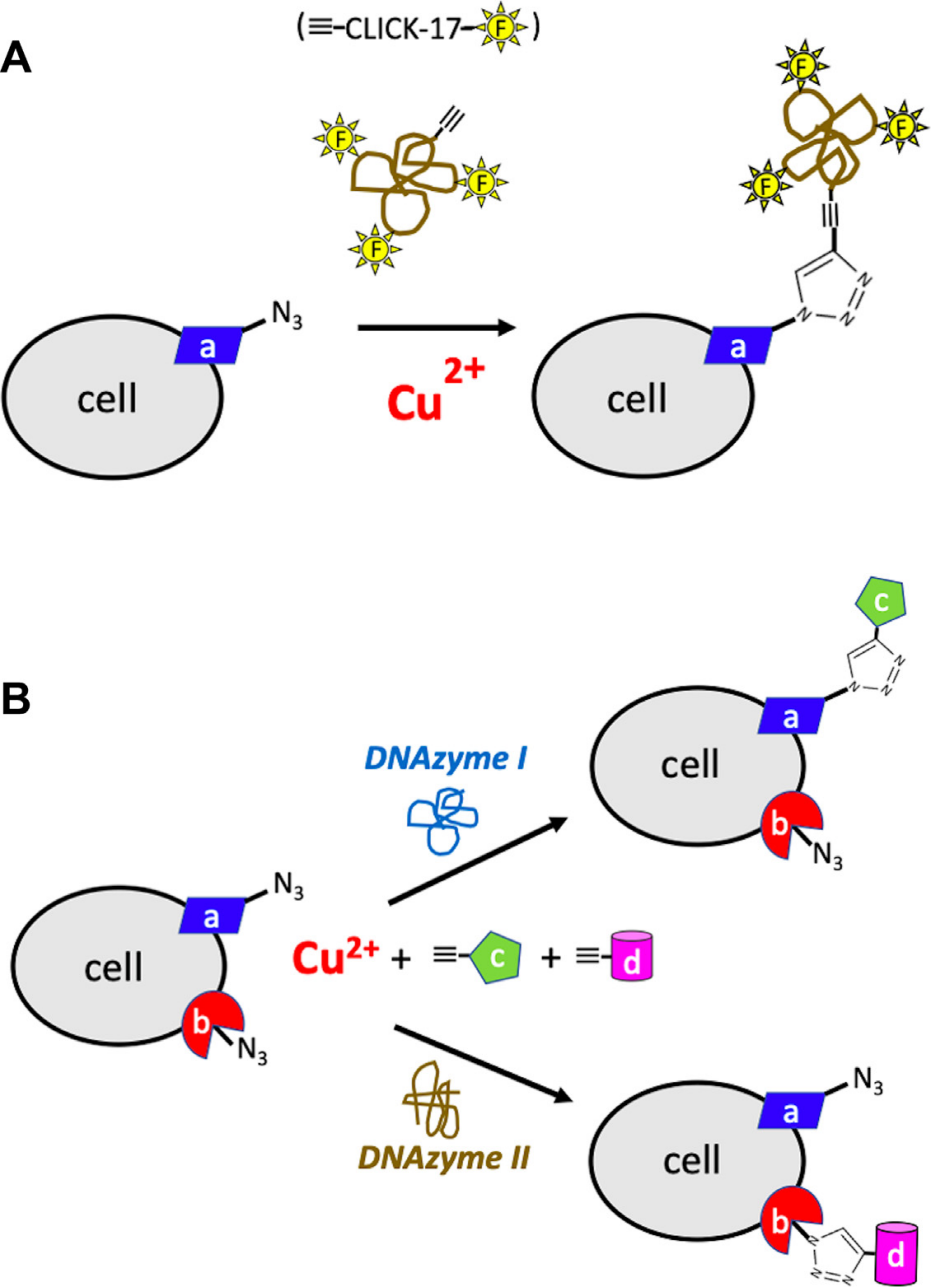
Two modes of predicted usefulness for Cu^2+^-utilizing, CuAAC-catalyzing DNAzymes. (**A**) The usefulness of *in cis* conjugation of the existing ≡-CLICK-17 DNAzyme, with covalently appended multiple fluorescent (as well as other) labels, to an azide-incorporating cell surface marker, ‘a’. (**B**) Orthogonal labeling cell surface determinants. In this schematic, the high substrate specificity that DNA enzymes are known to be capable of, is invoked. Here, a substrate-specific ‘DNAzyme I’ would specifically couple an azide-derivatized cell surface determinant (‘a’) with an alkyne-labeled tag, ‘c’, but not with an azide-derivatized cell surface determinant (‘b’) with an alkyne-labeled tag, ‘d’. Conversely, a different enzyme, ‘DNAzyme II’ would exclusively couple an azide-derivatized cell surface determinant (‘b’) with alkyne-‘d’ and not ‘a’ with ‘c’.

First, a stretch of DNA such as CLICK-17, synthesized in an automated DNA synthesizer, is easily derivatized (during synthesis) with multiple non-DNA functionalities, including either a variety or multiple copies of fluorophores, for instance. Given the high efficiency and very low concentration of either Cu(I) or Cu(II) required for the *in cis* reaction catalyzed by CLICK-17 (whereby ≡-CLICK-17 itself becomes attached to an azide-containing protein), for instance, a very high level of fluorophore and/or other functional group attachment to the protein should be possible. Figure 9 a illustrates this concept. We have shown that azido-lysozyme, as a model protein (containing four disulfide linkages with potential copper-sequestering ability) can be efficiently labeled with multiple fluoresceinated CLICK-17 DNAs (Figure 5) at very low μM Cu^2+^ (or Cu^+^) concentrations.

It is anticipated that we will be able, using systematic deletion mutagenesis, to reduce the effective chain length of CLICK-17, by eliminating sequences that are extraneous to the catalytic sequence motifs present within the 79-nt CLICK-17.

Figure 9B shows another of our conceptions of the potential usefulness of substrate-specific CuAAC DNAzymes, whereby different substrate-specific (as well as low Cu^2+^concentration-utilizing) DNAzymes could be used *orthogonally*. Thus, a ‘DNAzyme 1’ could substrate-specifically couple, say, an azide-labeled cell surface determinant, ‘a’, with an alkyne-linked tag, ‘c’, exclusively. Whereas, a different DNAzyme, ‘DNAzyme II’, could couple a different cellular determinant, ‘b’, exclusively, with a tag, ‘d’ in the same reaction mixture. Selecting for such highly substrate-specific ‘click’ DNAzymes and ribozymes is a key goal for our own research.

It is our expectation that DNAzymes such as CLICK-17 may present a number of advantages over small molecule catalysts, such as THPTA, within a defined but restricted set of applications (such as operating in an aqueous medium and not organic solvents) yet presenting key benefits. Both the DNAzymes themselves and their catalytic activity should be highly biocompatible (not least owing to the use of Cu^2+^ instead of Cu^+^, and at such low concentrations as to be minimally injurious to cells). Thus, they should be particularly useful for CuAAC labeling of the surfaces of live cells as well as for labeling viral coats (such as on bacteriophages used for phage display). A major advantage we foresee for the utilization of very low—10–20 μM—concentrations of Cu^2+^ (and not Cu^+^ generated *in situ* by a strong reductant such as ascorbate), is lower production (and lower concomitant damage from) ROS species. Being a macromolecule, the DNAzyme is furthermore likely to act as a superior sacrificial scavenger itself for any level of ROS species that may still be generated.

## Supplementary Material

gkaa502_Supplemental_File
