# Supplementary material for: CLICK-17, a DNA enzyme that harnesses ultra-low concentrations of either Cu+ or Cu2+ to catalyze the azide-alkyne ‘click’ reaction in water
Source: Nucleic Acids Res. 2020 Jun 10;48(13):7356–70. doi: 10.1093/nar/gkaa502 (PMC7367168; doi:10.1093/nar/gkaa502)
Supplement: gkaa502_Supplemental_File [file gkaa502_supplemental_file.pdf]

CLICK-17, a DNA Enzyme that Harnesses Ultra-Low Concentrations of either Cu^{+} or Cu^{2+} to Catalyze the Azide-Alkyne "Click" Reaction in Water

Kun Liu[1], Prince Kumar Lat[2], Hua-Zhong Yu[1,2*], and Dipankar Sen[1,2*]
[1]Dept. of Chemistry and [2]Dept. of Molecular Biology & Biochemistry
Simon Fraser University
Burnaby, BC V5A 1S6, Canada

Supporting Information

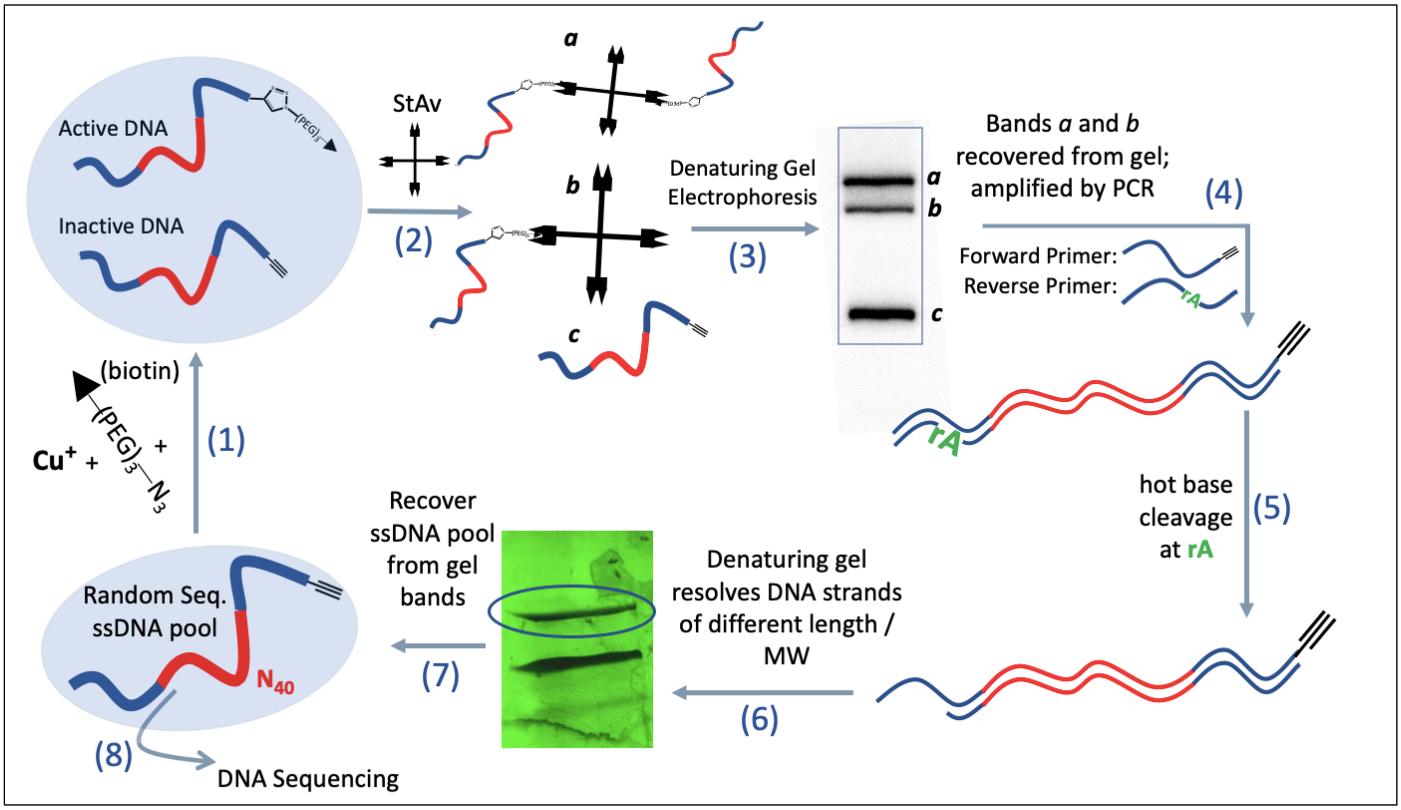

<u>Fig. S1</u>: Schematic diagram for the SELEX scheme used in this work. (1) Reaction of a folded, random sequenced ("N_{40}" within 80 nt) 5'-hexynyl single-stranded DNA (ssDNA) pool/library with azide-biotin (the black arrowhead represents the biotin moiety) and *in situ* generated Cu^+; (2) separation of individual sequences of CuAAC-coupled 5'-hexynyl ssDNAs from unreacted DNAs in the library by binding to streptavidin ("StAv") followed by (3) denaturing (or native) gel electrophoresis; (4) purification from the gel of StAv-retarded DNA bands, and their PCR amplification using a 5'-alkynated forward DNA primer and a reverse DNA primer incorporating a single internal adenine riboside ("rA"). (5) Hot-base cleavage of the ribose-containing DNA strand within the PCR-generated duplex; (6) size-separation and purification of the alkynylated ssDNA from the non-alkynylated ssDNA; and (7) repetition of the selection cycle, or sequencing of the DNA pool (8) to identify individual DNA sequences that promote CuAAC.

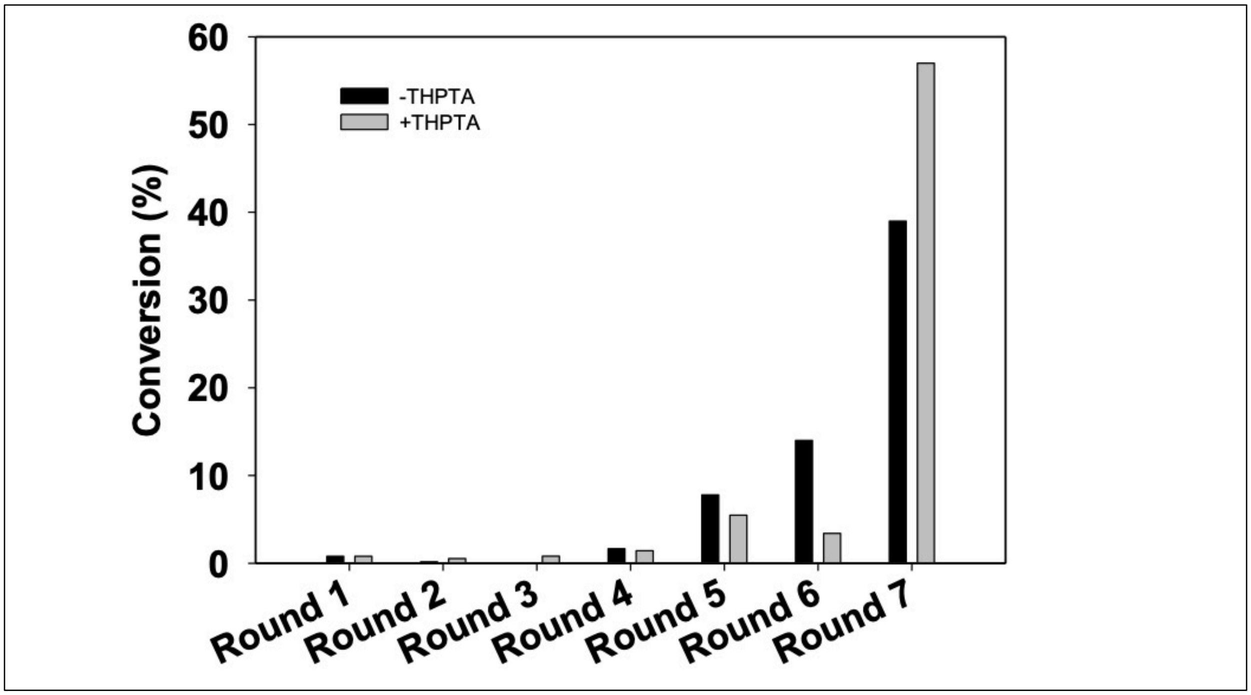

<u>Fig. S2</u>: **Enrichment of *in cis* CuAAC-catalyzing DNAzymes from the first seven rounds of *in vitro* selection.** Enrichment percentages of the evolving random-sequence DNA library is plotted as a function of selection round, following the selection protocol shown in **Fig. 1**.

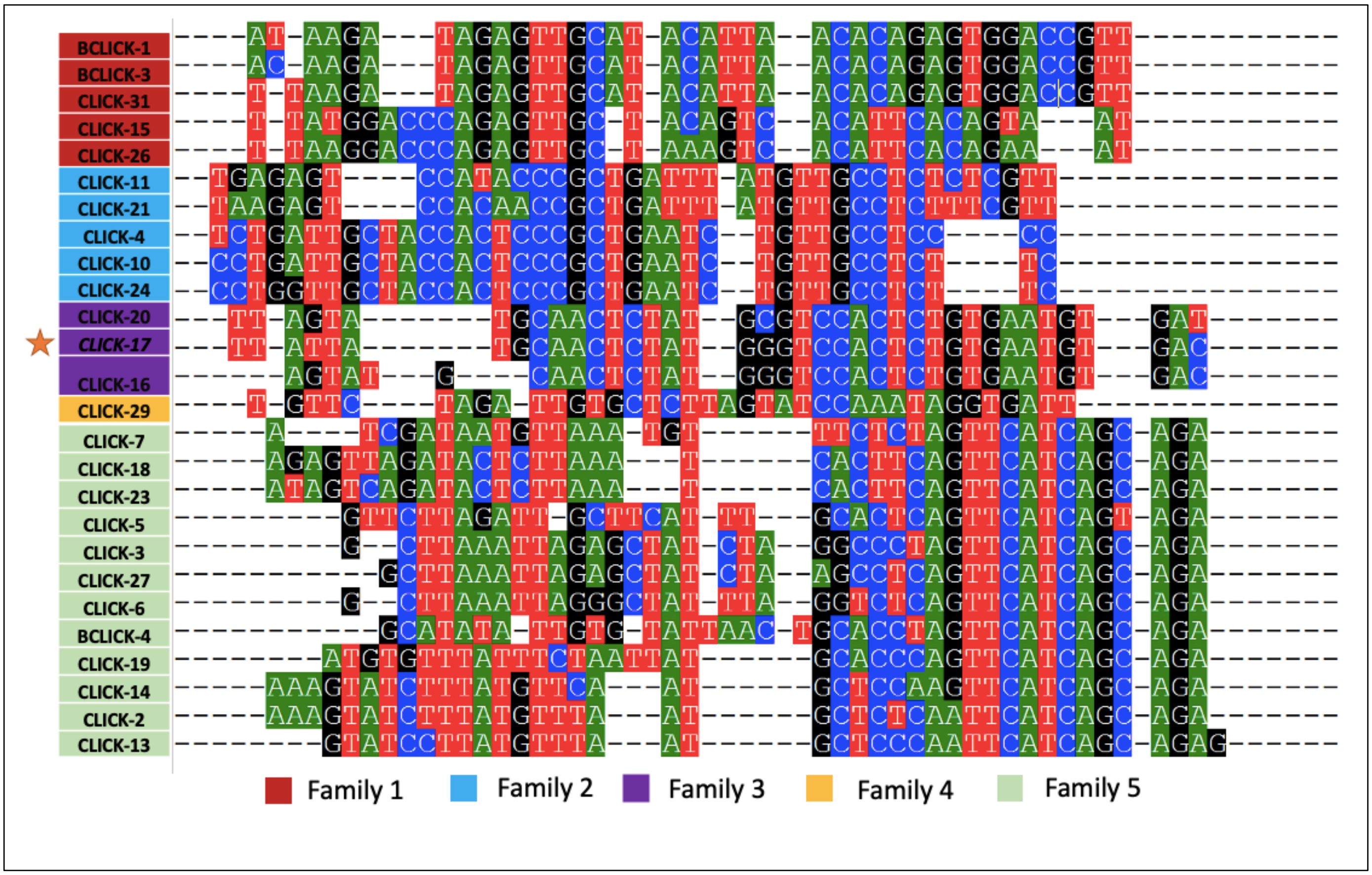

<u>**Fig. S3**</u>**: The N_{40} regions of cloned DNA sequences.** 40 clones from Round 25 of the *in vitro* selection experiment are shown. Some of the sequences shown above were present in multiple clones. The clone CLICK-17, used for the bulk of this study, is shown with an asterisk.

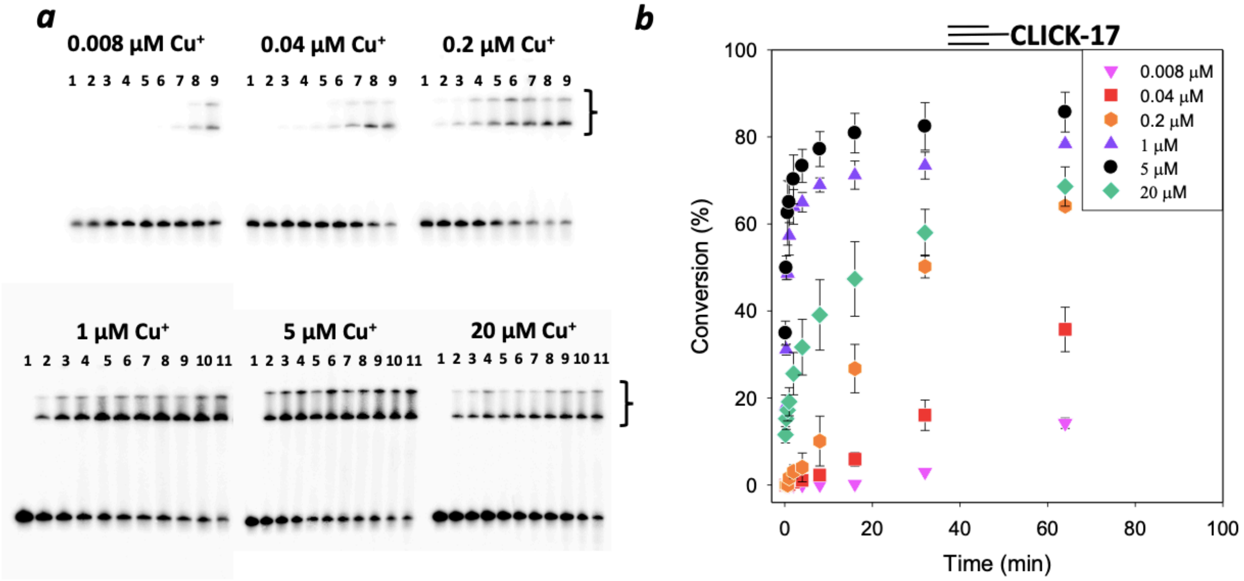

<u>Fig. S4</u>: Kinetics for ≡-CLICK-17 biotinylation via CuAAC as a function of Cu^{+} concentration. ≡-CLICK-17 DNA concentration was fixed at 2 μM. For the upper panel of three gels, lanes 1-9 show reaction times of: 0 min; 1; 2; 4; 8; 16; 32; 64; and 128 min, respectively. In the lower panel of three gels, lanes 1-11 show: 0 s; 10 s; 20 s; 40 s; then, 1; 2; 4; 8; 16; 32; and 64 min. The error bars report one standard deviation from two independently collected data sets.

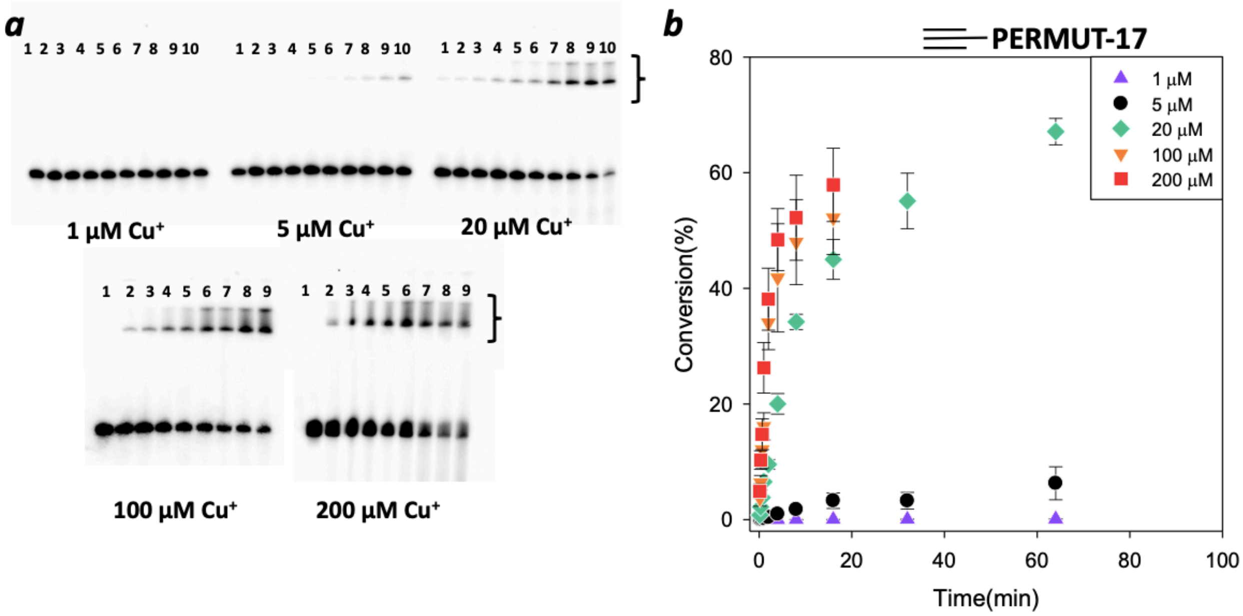

Fig. S5: **Kinetics for ≡-PERMUT-17 biotinylation via CuAAC as a function of Cu^+ concentration.** ≡-PERMUT-17 DNA concentration was fixed at 2 µM. For the upper panel of three gels, lane 1-9 show reaction times of: 0 min; 1; 2; 4; 8; 16; 32; 64; and,128 min. In the lower two gels, lanes 1-11 show: 0 s; 10 s; 20 s; 40 s; 1 min; 2; 4; 8; 16; 32; and 64 min reactions. The error bars report one standard deviation from two independently collected data sets.

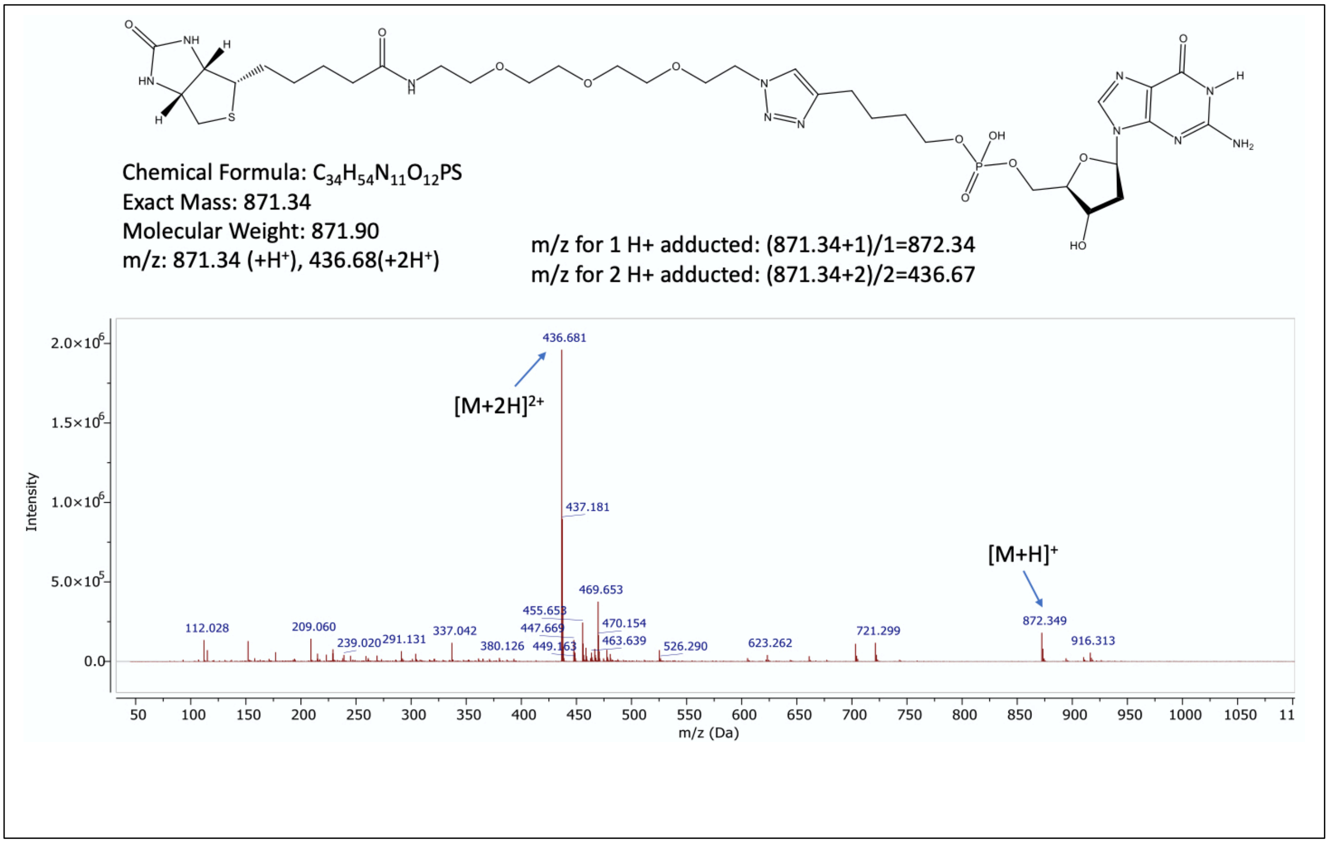

Fig. S6: ESI Mass spectrometric analysis of the *in cis* conjugation of ≡-CLICK-17 with azide-biotin. The presumed conjugation product was first treated with Endonuclease P1, which degrades single-stranded DNA to 5'-deoxyribonucleotide monophosphates. Complete digestion of the conjugated product would leave a residual structure that incoporates the 5'-most deoxyriboguanosine monophosphate residue of the CLICK-17 DNA sequence linked to azide-biotin via the triazole and linker atoms as shown.

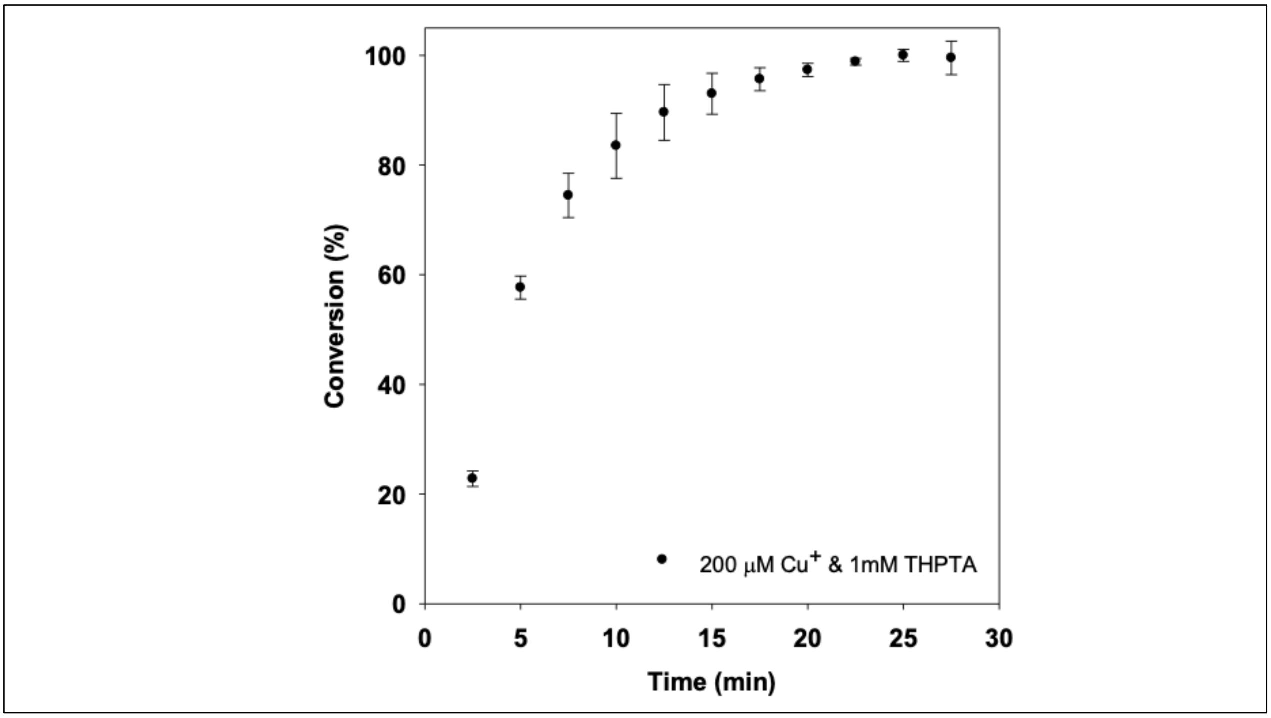

<u>Fig. S7</u>: Fluorescence calibration curve. Calibration curve for the CuAAC conversion of 50 μM azide-biotin using 2 mM propargyl alcohol in 50 mM Li-HEPES, pH 7.4; and 200 μM $CuSO_4$ in the presence of 2.5 mM sodium ascorbate and 1 mM THPTA. The DNA and the substrates were mixed in a reaction tube, and the reaction initiated with the addition of ascorbate and THPTA to a final reaction volume of 40 μl. The solution was quickly transferred into a 384-well plate for fluorescence measurement in an Infinite M200 Pro (Tecan) fluorescence plate reader. The error bars report one standard deviation from three independently collected data sets.

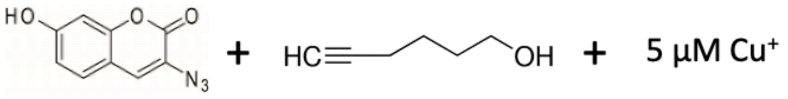

a

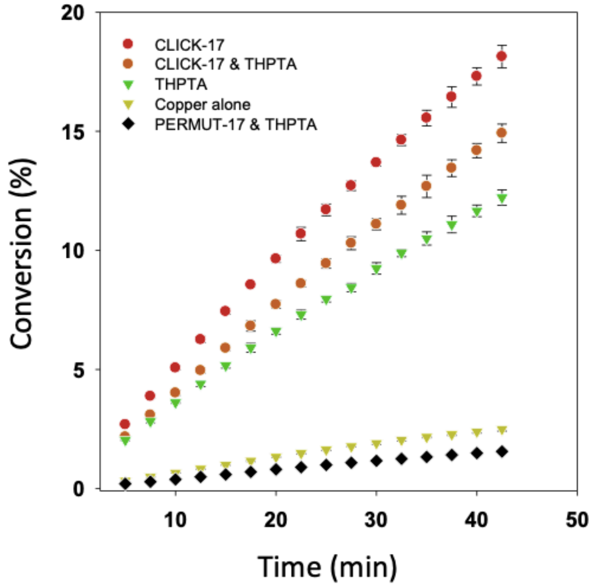

b

	Initial rate (µM/min)
2 µM CLICK-17	0.24
2 µM CLICK-17 + 25 µM THPTA	0.19
25 µM THPTA	0.16
Cu^+ alone	0.03
2 µM PERMUT-17 + 25 µM THPTA	0.02
2 µM PERMUT-17	< 0.01

Fig. S8: CLICK-17 is capable of utilizing Cu^+ to catalyze CuAAC *in trans* between freely diffusing hexynol and azide-coumarin substrates. The conditions of reaction consisted of 50 µM 3-azido-7-hydroxycoumarin ("azide-coumarin"), 8 mM hexynol, 5 µM Cu^+, and the different combination of catalysts and reagents shown in the Table. Reactions were started by the addition of sodium ascorbate. The error bars report one standard deviation from three independently collected data sets.

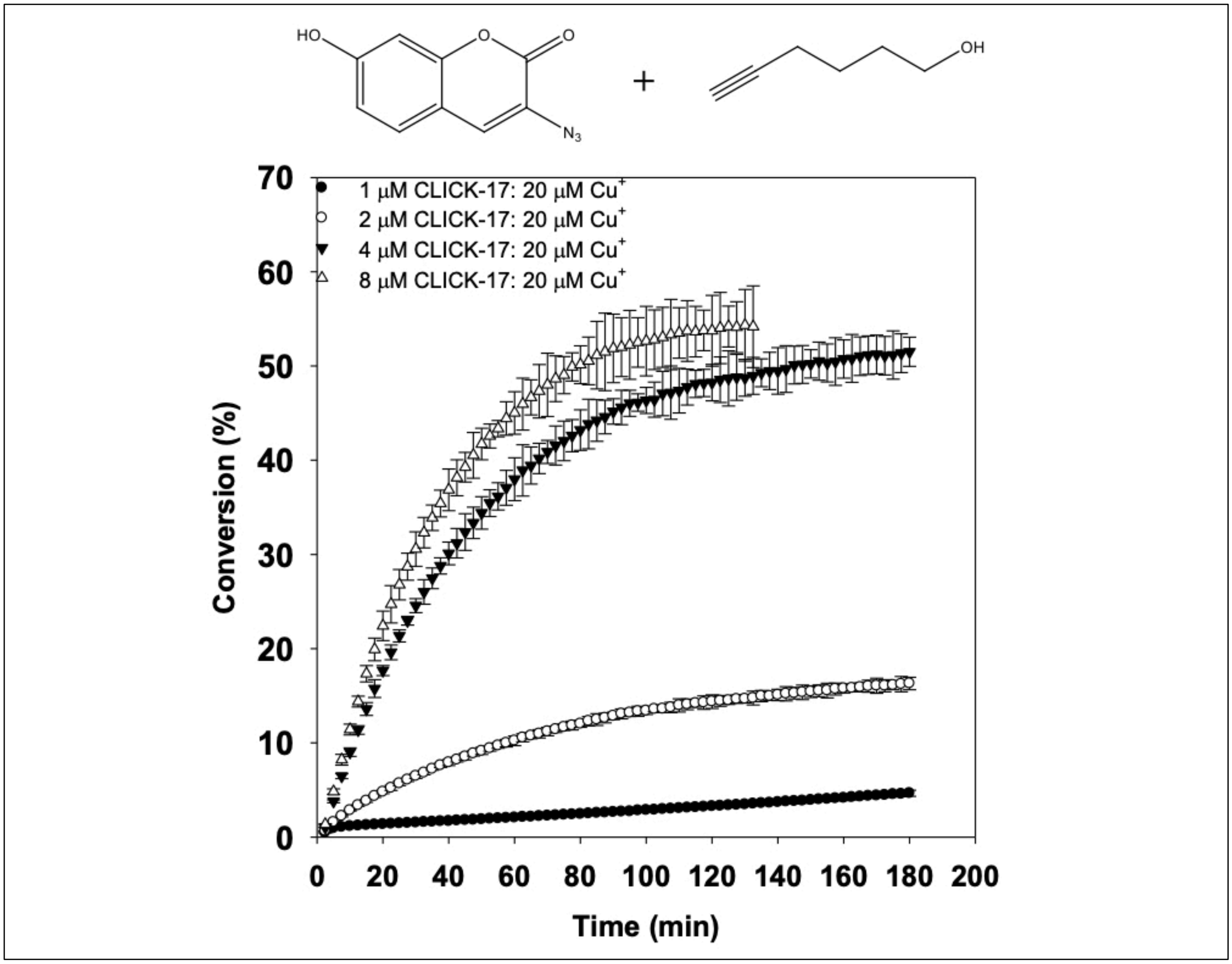

<u>Fig. S9</u>: The impact of CLICK-17 DNA concentrations on the *in trans* CuAAC reactions catalyzed by CLICK-17. Fixed concentrations of azide-coumarin (50 µM), hexynol (8 mM), and Cu^+ (20 µM) were used in R buffer, at 22 °C, with CLICK-17 DNA concentration varying, as above. The error bars report one standard deviation from three independently collected data sets.

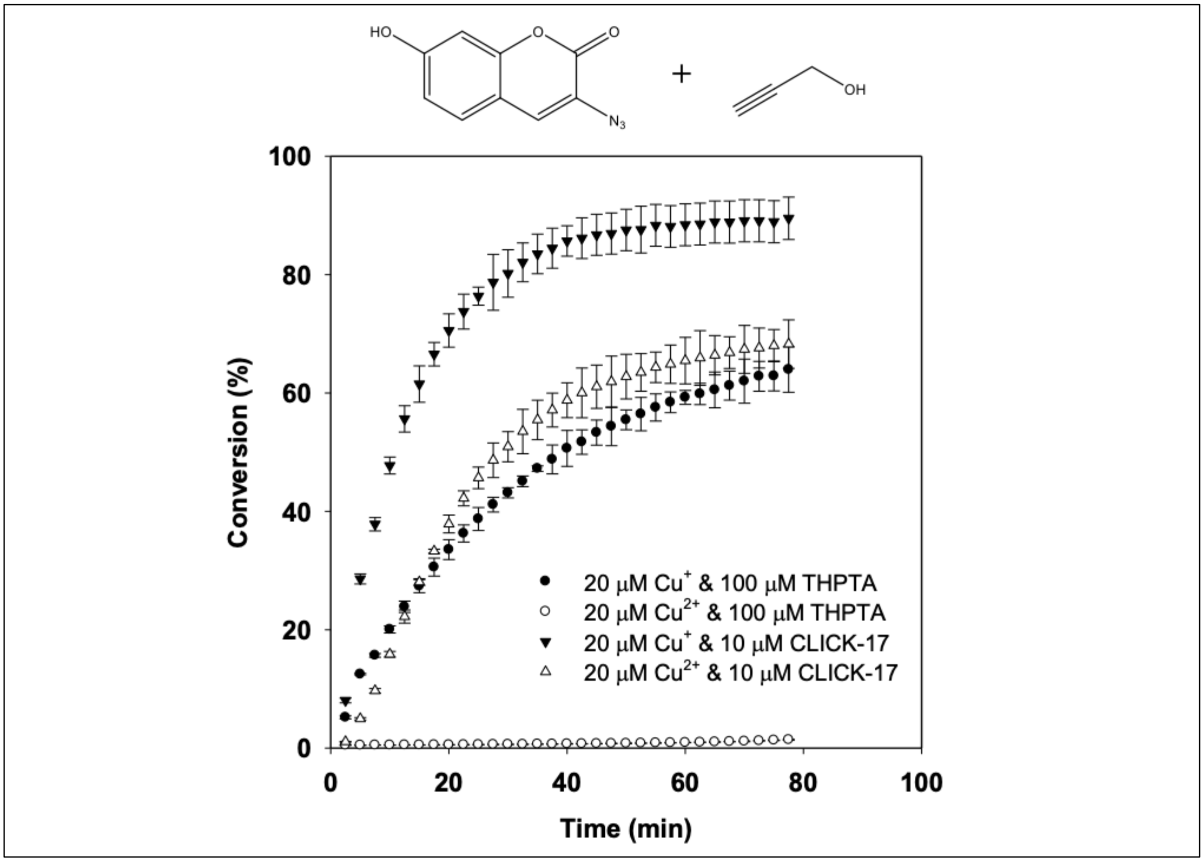

<u>Fig. S10</u>: CLICK-17 catalysis with Cu^{+} and Cu^{2+} under optimized conditions. Catalysis *in trans* for quantitative conversion to product was achieved within 40 min in the presence of Cu^{+} (20 μM Cu^{+}, 10 μM CLICK-17, 50 μM azide-coumarin, 20 mM propargyl alcohol) at 22 °C in R buffer. A comparison yield at 40 min with 100 μM THPTA is ~ 55%. In the presence of Cu^{2+}, however, yield over 40 min with 10 μM CLICK-17 is ~60%, while with 100 μM THPTA, only ~2%. In these experiments, azide-coumarin is the limiting subsrate; and with 20 mM propargyl alcohol CuAAC is ~90% complete within 40 min at 22 °C, whereas with 100 μM THPTA ligand replacing CLICK-17, only ~60% of the reaction is completed even after 80 min. The use of Cu^{2+} instead of Cu^{+} gives an even more striking result, with > 60% reaction completion in 80 min with CLICK-17 and *no* reaction on this timescale with THPTA replacing CLICK-17. The error bars report one standard deviation from three independently collected data sets.

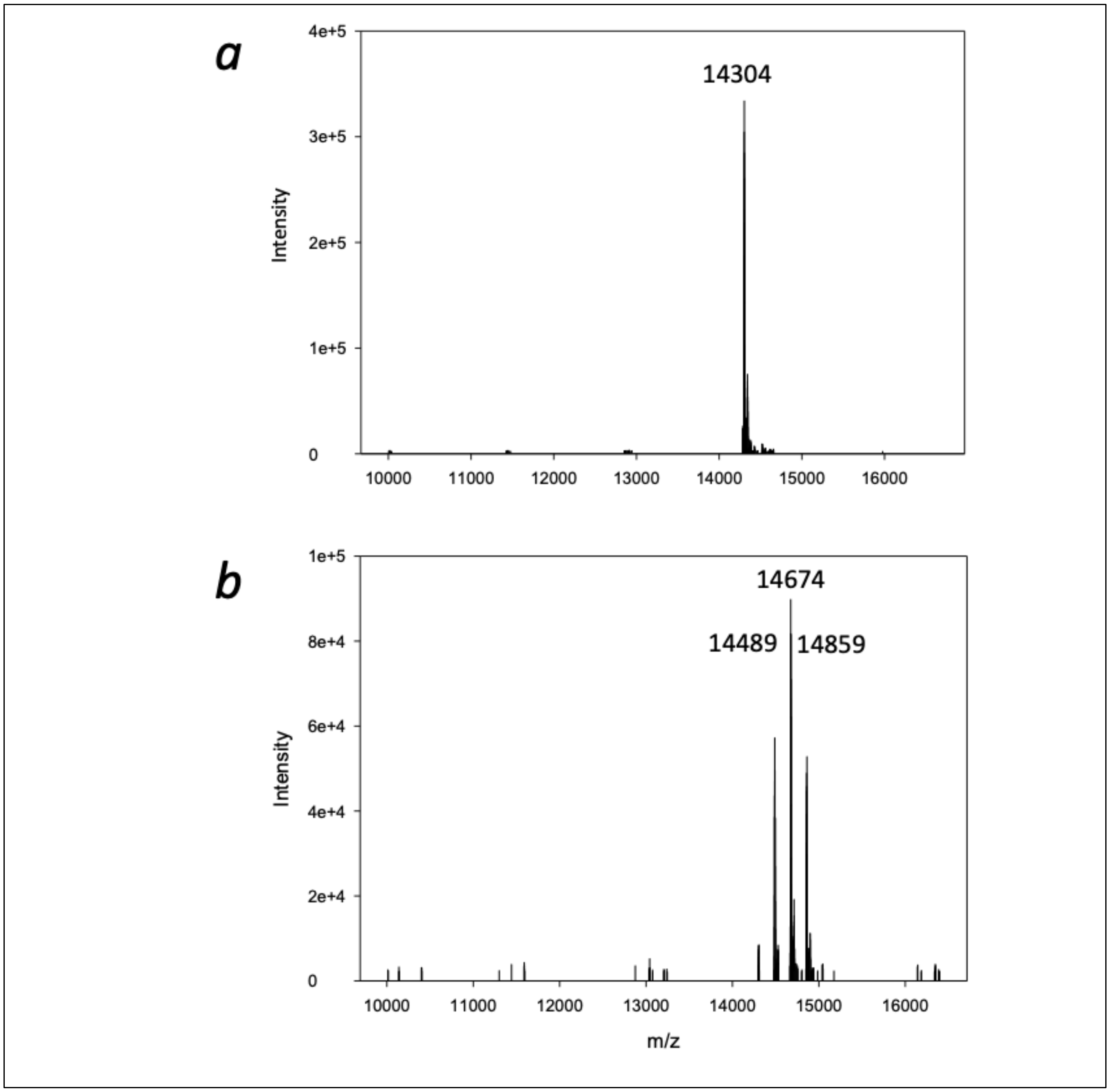

<u>Fig. S11</u>: ESI mass spectrometry analysis of the azide labeling of lysozyme. ***Top***, lysozyme protein whole protein LC-mass spectrometry data (ESI). The peak at 14304 Da is consistent with molecular weight of lysozyme. ***Bottom***, lysozyme labelled with N3-PEG2-NHS ester. The peak at 14303 decreased dramatically, and three new peaks appears at 14489, 14674, 14859, which correspond to lysozyme with 1, 2, and 3 N3-PEG2 labels, respectively (each label adds 185 Da to the protein).

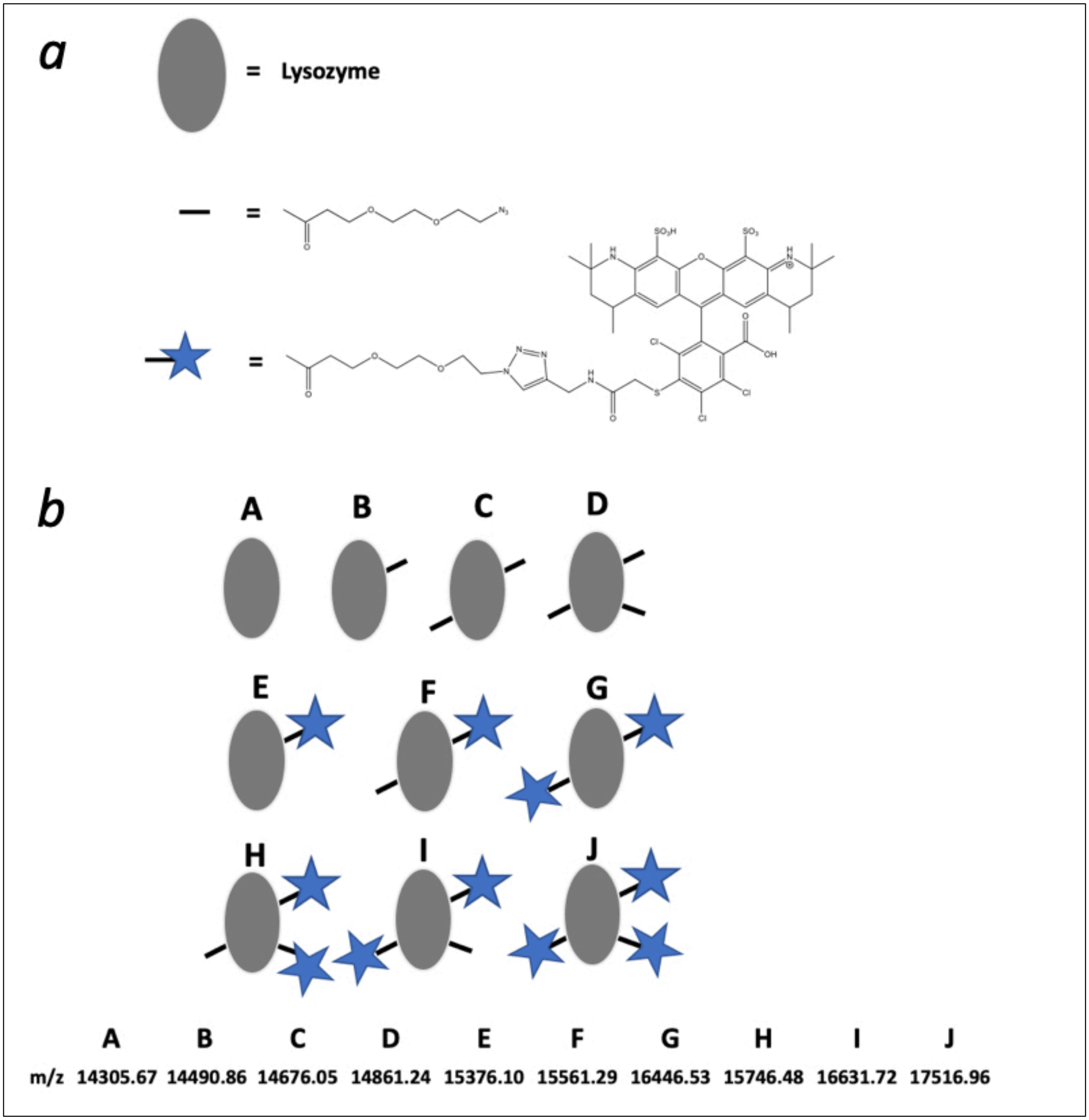

Fig. S12: (***a, b***) **Schematic of symbols used for the ESI mass spectrometry data shown in Supplementary Fig. S13**. Product A represents unmodified lysozyme; B-D are lysozyme-$(N_3)_{1-3}$; whereas, E-J represent different conjugates of lysozyme-$(N_3)_{1-3}$ with ≡-AFDye 546.

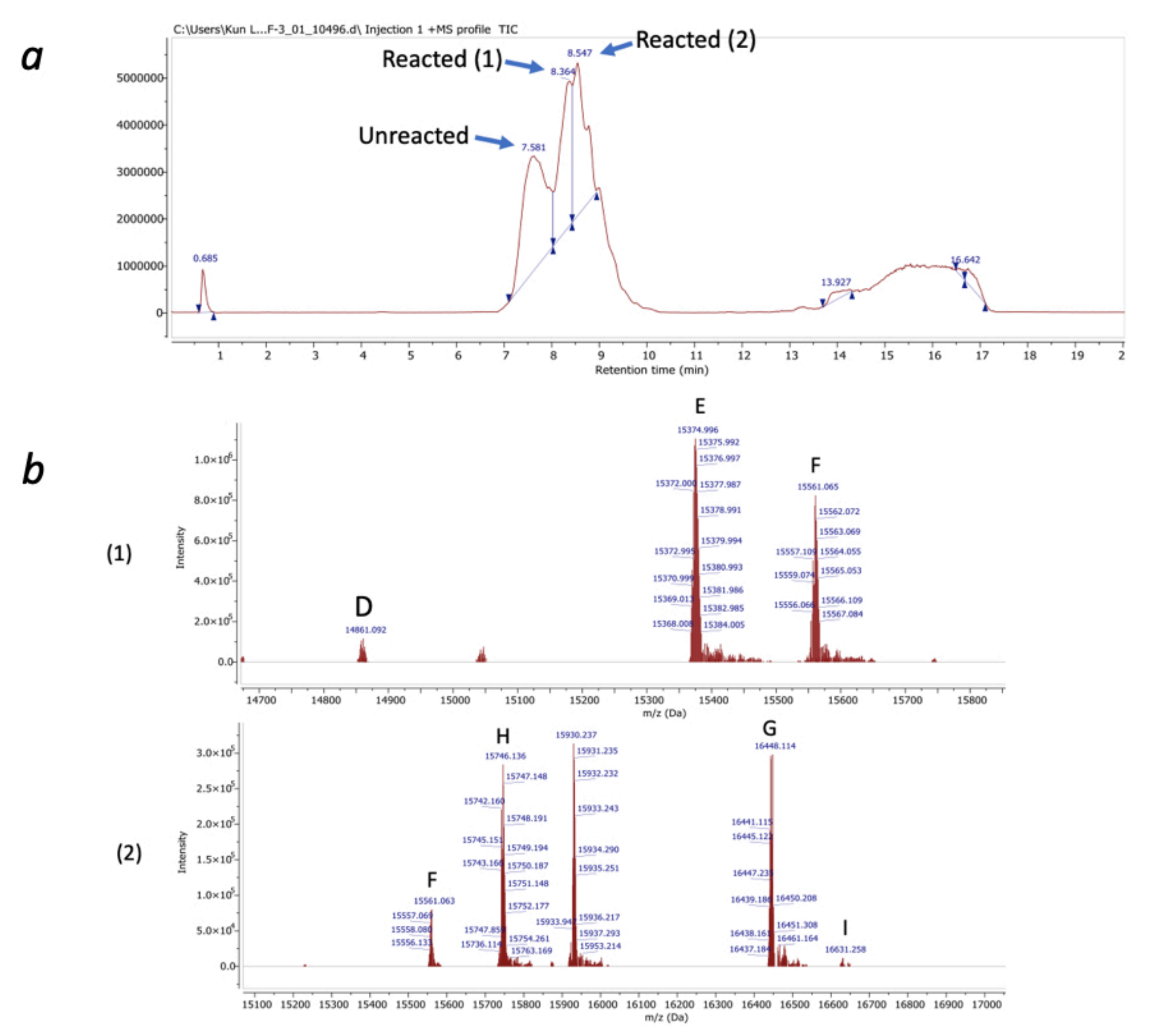

Fig. S13: (***a***) **LC Chromatogram**, showing two peaks of interest, "1" and "2", with retention times of 8.37 min, and 9.01 min, respectively; (***b***) **m/z profiles** of chromatogram peaks "1" and "2", representing different lysozyme-$(N_3)_{1\text{-}3}$ and ≡-AFDye 546 conjugates (products D-I shown, defined in Supplementary Fig. 11).

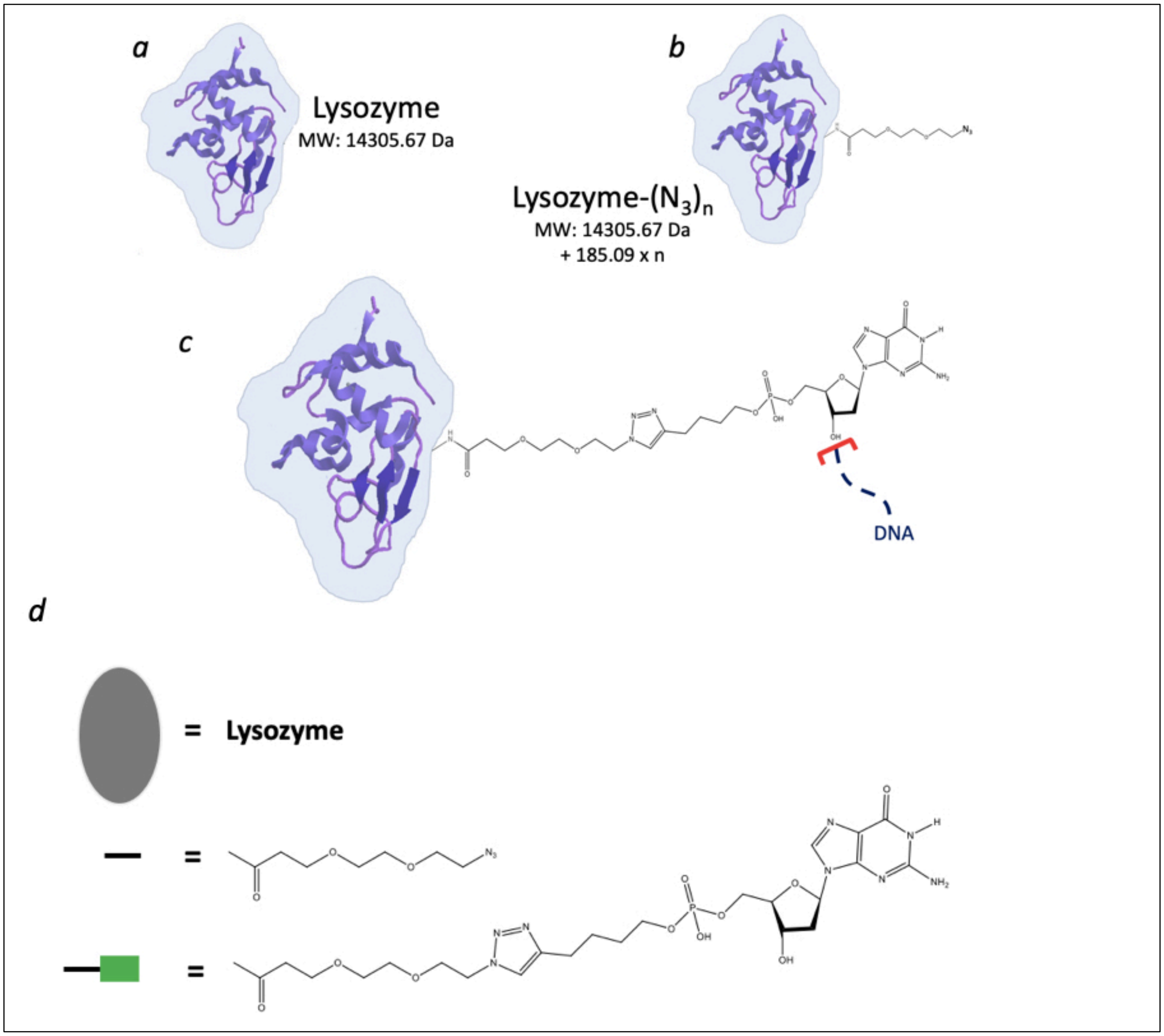

<u>Fig. S14</u>: Expected molecular weights of underivatized lysozyme (***a***), and of azide-containing lysozyme-$(N_3)_n$ (***b***), are shown. (***c***) Schematic diagram of lysozyme-$(N_3)_n$ (where n = 1), and the *in cis* conjugated product of lysozyme-$(N_3)_n$ (where n = 1) reacted with ≡-CLICK-17 (the full length of the conjugated CLICK-17 is shown as "DNA"). For the purpose of ESI mass spectrometry analysis, the DNA was total-digested with Nuclease P1, an endonuclease that generates 5'-deoxyribonucleotide monophosphates. The portion of the appended CLICK-17 DNA removed by the nuclease P1 is shown by the red bracket. The expected structure of the DNA residue, incorporating the 5'-most deoxyriboguanosine of the CLICK-17 nucleotide sequence, is shown. (***d***) Schematic of symbols used for the ESI mass spectrometry data shown in Supplementary Fig. S15.

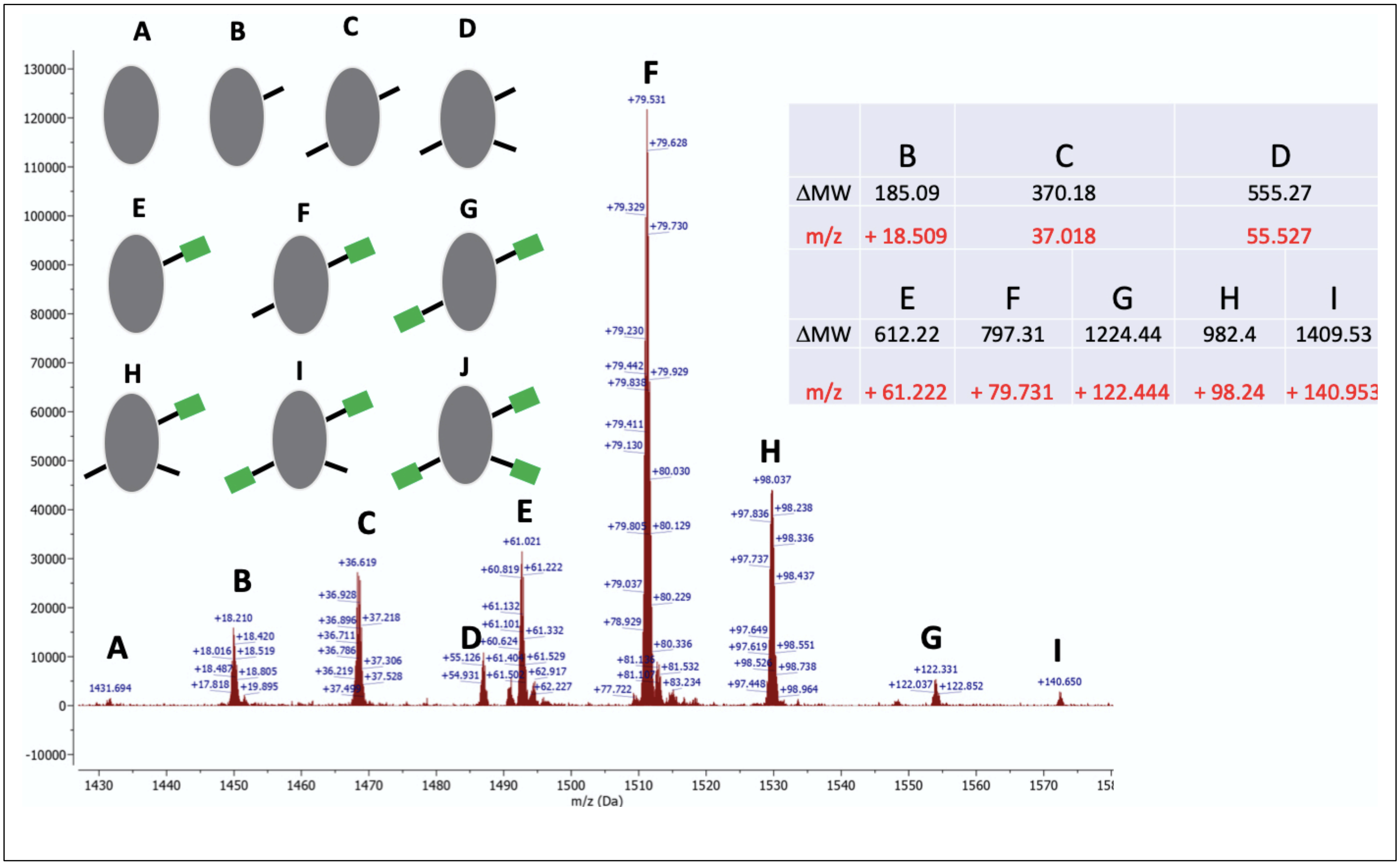

<u>Fig. S15</u>: ESI mass spectrometry data obtained from the Cu$^+$-dependent and CLICK-17-catalyzed *in cis* conjugation of lysozyme-$(N_3)_{1\text{-}3}$ with ≡-CLICK-17 itself. The various DNA-protein conjugate species formed (see Supplementary Figure S12 for an explanation for the symbols used) were treated with Nuclease P1 to digest away almost the entirety of each conjugated DNA in each case, leaving only the 5'-most deoxyriboguanosine residue behind as a fingerprint of the original DNA attachment.

CLICK-17: GGATC GTCAG TGCAT TGAGA TTATT ATGCA ACTCT ATGGG TCCAC TCTGT GAATG TGACG GTGGT ATCCG CAACG GGTA

CL-17_T24G: GGATC GTCAG TGCAT TGAGA TTAGT ATGCA ACTCT ATGGG TCCAC TCTGT GAATG TGACG GTGGT ATCCG CAACG GGTA

CLICK-16: GGATC GTCAG TGCAT TGAGA - -AGT ATGCA ACTCT ATGGG TCCAC TCTGT GAATG TGACG GTGGT ATCCG CAACG GGTA

CLICK-20: GGATC GTCAG TGCAT TGAGA TTAGT ATGCA ACTCT ATGCG TCCAC TCTGT GAATG TGATG GTGGT ATCCG CAACG GGTA

CL-20_C39G: GGATC GTCAG TGCAT TGAGA TTAGT ATGCA ACTCT ATGGG TCCAC TCTGT GAATG TGATG GTGGT ATCCG CAACG GGTA

CL-20_G24T: GGATC GTCAG TGCAT TGAGA TTATT ATGCA ACTCT ATGCG TCCAC TCTGT GAATG TGATG GTGGT ATCCG CAACG GGTA

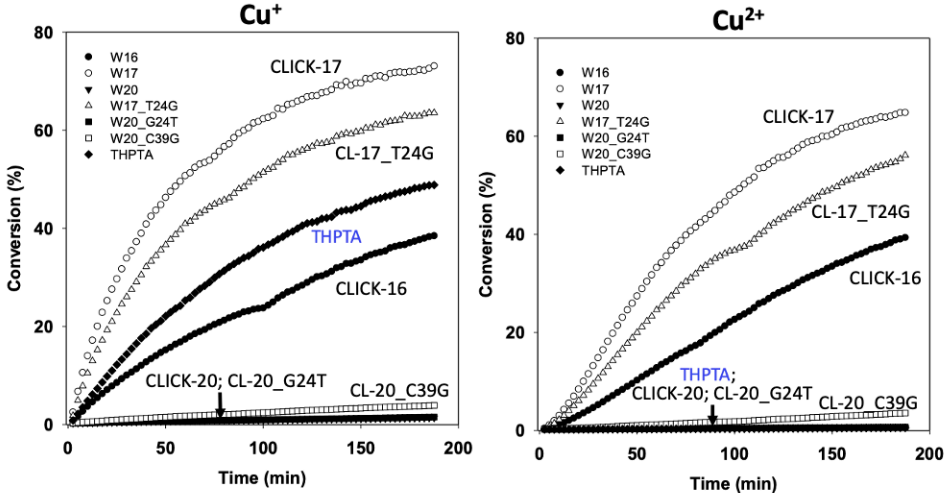

Fig. S16: *Above,* Nucleotide sequences of three of the original DNA clones (CLICK-17, CLICK-16, and CLICK-20) obtained from the initial SELEX experiment for DNAzymes catalytic for the CuAAC reaction, as well as nucleotide sequences of three mutants, CLICK-17_T24G, CLICK-20_C39G, and CLICK-20_G24T. *Below*, Cu^{+}-dependent (*left*) and Cu^{2+}-dependent *in trans* conjugation profiles of hexynol with azide-coumarin.

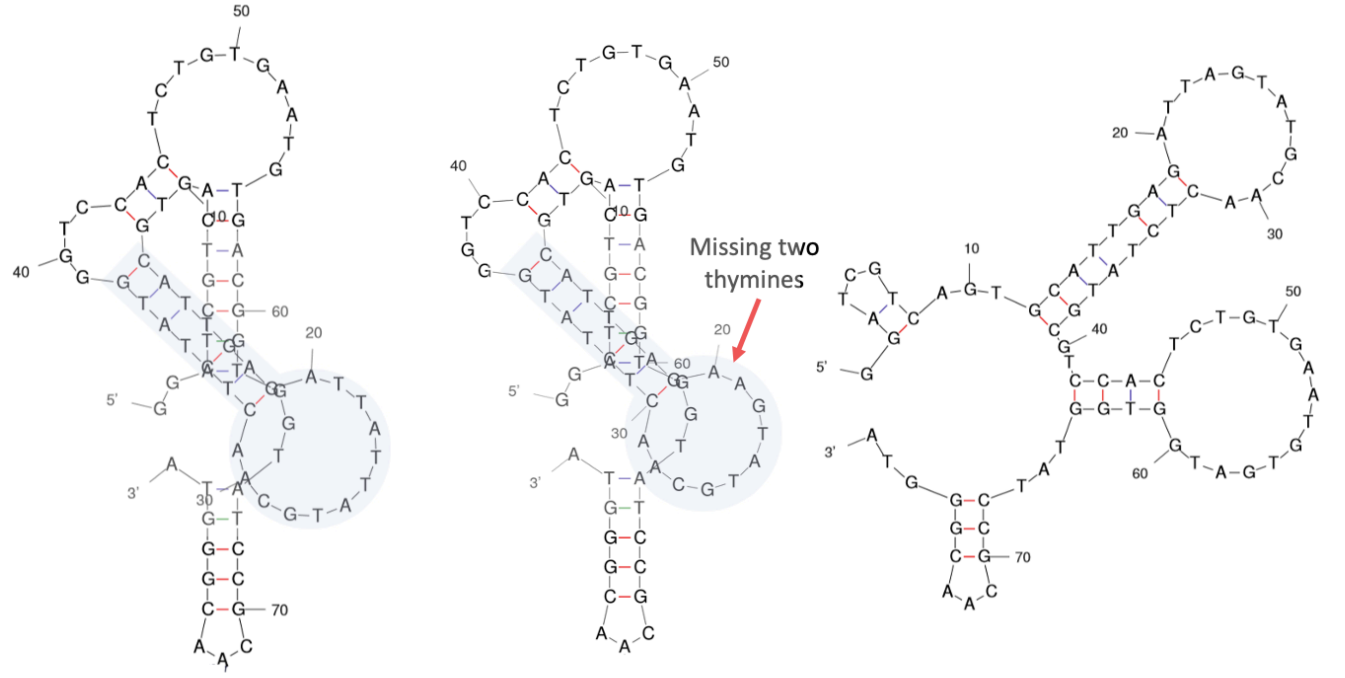

CLICK-17
ΔG = -19 kcal/mol

CLICK-16
ΔG = -18 kcal/mol

CLICK-20
ΔG = -18 kcal/mol

<u>Fig. S17</u>: **Secondary structure predictions**, based on the Mfold program, for the the DNA sequences of clones CLICK-17, CLICK-16, and CLICK-20. The most thermodynamically stable predicted structure for each clone is shown.

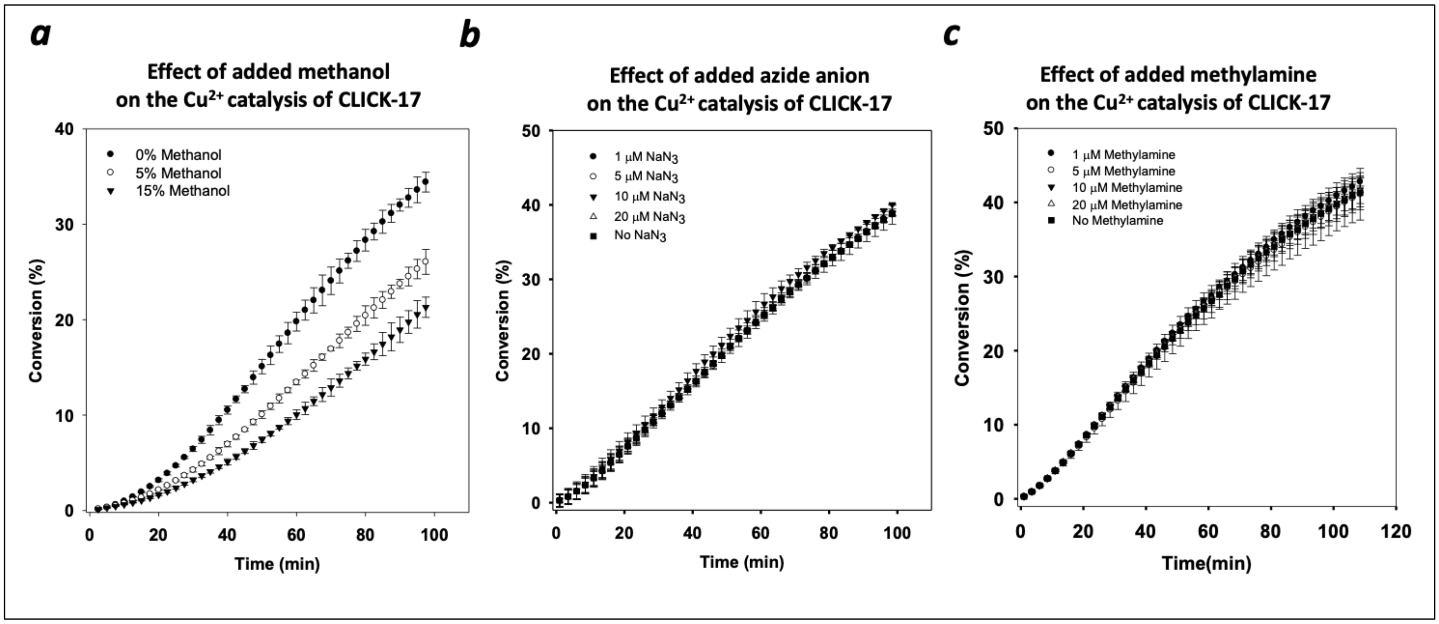

<u>**Fig. S18**</u>: **Effect of supplementations of methanol; azide anion; and methylamine on CLICK-17's "Cu^{2+}" catalysis**. The data, above, plot averages from three independent experiments carried out for each of the supplementations shown. The error bars for the methanol data report one standard deviation from two independently collected data sets. Error bars for the azide anion and methylamine data report one standard deviation from three independently collected data sets.

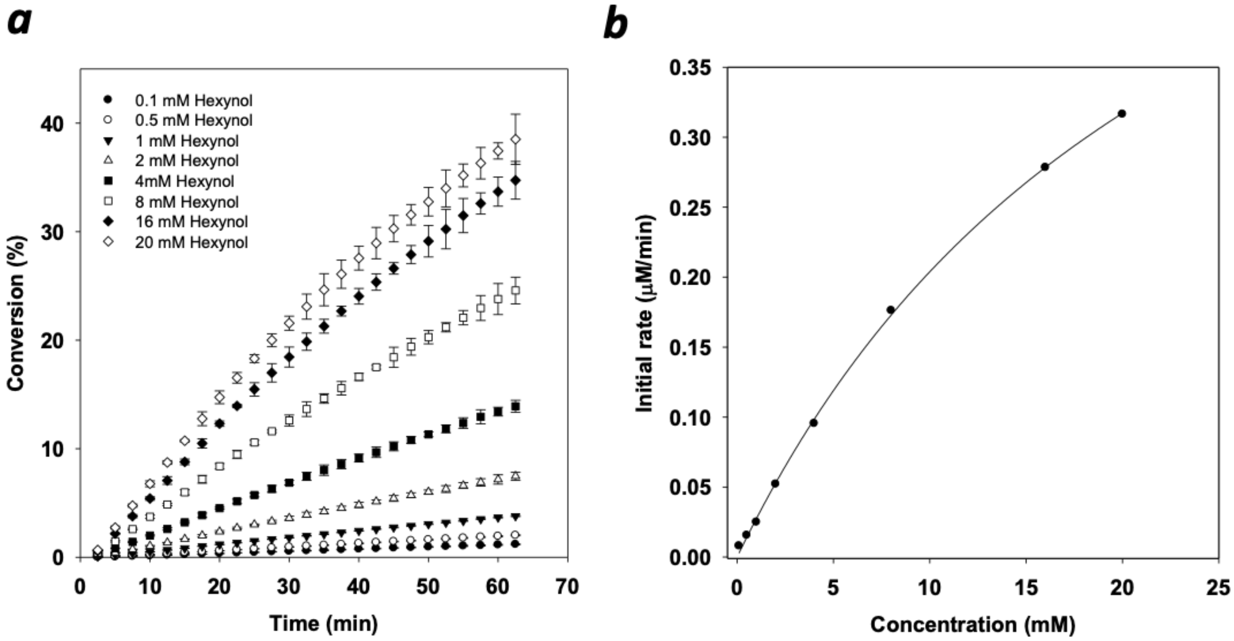

<u>Fig. S19</u>: Does folded CLICK-17 supply a high-affinity binding site for hexynol? Reaction time-dependence profiles of 2 μM CLICK-17 DNA catalyzing the reaction of 5-hexyne-1-ol ("hexynol"; 0.1-20 mM) in R buffer, with a fixed concentration of 3-azido-7-hydroxycoumarin ("azide-coumarin"; 50 μM) and Cu^{+} (5 μM). The error bars report one standard deviation from three independently collected data sets.
